# BROAD‐NESS Uncovers Dual‐Stream Mechanisms Underlying Predictive Coding in Auditory Memory Networks

**DOI:** 10.1002/advs.202507878

**Published:** 2025-09-29

**Authors:** Leonardo Bonetti, Gemma Fernández‐Rubio, Mathias H. Andersen, Chiara Malvaso, Francesco Carlomagno, Claudia Testa, Peter Vuust, Morten L. Kringelbach, Mattia Rosso

**Affiliations:** ^1^ Center for Music in the Brain Department of Clinical Medicine Aarhus University & The Royal Academy of Music, Aarhus/Aalborg Denmark; ^2^ Centre for Eudaimonia and Human Flourishing Linacre College University of Oxford Oxford OX39BX United Kingdom; ^3^ Department of Psychiatry University of Oxford Oxford OX37JX United Kingdom; ^4^ Danish Research Centre for Magnetic Resonance Hvidovre 2650 Denmark; ^5^ Department of Physics and Astronomy University of Bologna Bologna 40126 Italy; ^6^ Department of Education Psychology Communication University of Bari Aldo Moro Bari 70121 Italy; ^7^ IPEM Institute for Systematic Musicology Ghent University Ghent 9000 Belgium

**Keywords:** brain networks, memory recognition, phase space, principal component analysis (PCA), spatial gradients

## Abstract

Whole‐brain network dynamics are yet to be fully understood, particularly in the context of auditory memory and predictive coding. BROAD‐NESS (BROADband brain Network Estimation via Source Separation), a novel pipeline for extracting broadband whole‐brain networks from source‐reconstructed MEG data is introduced. During auditory sequence recognition, BROAD‐NESS identified two orthogonal networks centered on auditory cortices. The first, also encompassing medial cingulate, is primarily involved in processing sounds and shows consistent but less marked differences between experimental conditions. The second, involving hippocampus, anterior cingulate, insula, and inferior temporal regions, exhibits strong condition‐dependent dynamics, reflecting engagement in confirmed predictions and prediction errors. The networks differ in temporal dynamics, spatial gradients, and behavioral relevance. Phase space and recurrence quantification analysis (RQA) reveal that more recurrent and stable dynamics are linked to higher accuracy and faster responses across sequence types. BROAD‐NESS also enables direct PCA versus ICA comparison, showing PCA‐based networks to be more robust and interpretable. Conceptually, this work reveals a dual‐stream embedded organization of auditory memory networks that mirrors, yet functionally diverges from, visual pathways. Methodologically, it introduces BROAD‐NESS, a powerful and interpretable pipeline for characterizing the spatiotemporal architecture of brain networks in neurophysiology.

## Graphical abstract

BROADband Network Estimation via Source Space (BROAD‐NESS) is a flexible analytical pipeline for identifying large‐scale brain networks from event‐related, source‐reconstructed MEG data, with a focus on auditory predictive and memory processes. BROAD‐NESS integrates a suite of complementary tools, including PCA‐based decomposition, phase space, and recurrence quantification analysis (RQA), spatial gradients embedding, and ICA comparisons, to extract and characterize functionally meaningful brain network dynamics. These integrated analyses underscore both the methodological robustness and neuroscientific utility of BROAD‐NESS in capturing temporally resolved, large‐scale neural activity patterns.

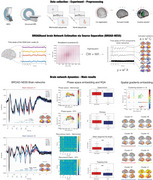

## 1 Introduction

Predictive coding is a fundamental principle of brain function, proposing that perception and cognition rely on hierarchical predictions to minimize discrepancies between expected and incoming sensory information.^[^ ^1^, ^2^, ^3^ ^]^ This framework has been extensively investigated in relation to automatic sensory processes, often indexed by event‐related potential/field (ERP/F) components such as N100, mismatch negativity (MMN), and error‐related negativity (ERAN).^[^ ^4^, ^5^, ^6^, ^7^, ^8^, ^9^, ^10^, ^11^, ^12^ ^]^ These studies have demonstrated that such components are automatically elicited in response to deviations in visual and auditory stimuli, including changes in expected image and sound features, variations in the probability of sound occurrence (N100, MMN),^[^ ^4^, ^5^, ^6^, ^7^, ^8^, ^9^, ^10^ ^]^ and musical harmonic properties (ERAN).^[^ ^11^, ^12^ ^]^ In addition, considerable research has explored conscious predictive coding, where individuals explicitly anticipate upcoming stimuli. Most studies in this domain have examined its neural correlates within decision‐making,^[^ ^13^ ^]^ memory‐based predictions,^[^ ^14^, ^15^ ^]^ or the interplay between conscious and automatic predictive processes.^[^ ^16^ ^]^

As in many areas of cognitive neuroscience, predictive coding research has also progressively transitioned from activation‐based approaches toward connectivity analyses. This shift was driven by mounting evidence that the brain operates as a complex system, where regions interact within distributed networks rather than functioning in isolation.^[^ ^17^, ^18^ ^]^ Previous research has used various metrics to assess functional connectivity, with dynamic causal modelling (DCM) emerging as the primary method for inferring connectivity between a limited set of brain regions in the context of predictive coding.^[^ ^19^ ^]^ For instance, DCM has been widely used to investigate the hierarchical organization of predictive coding during automatic processing, revealing the flow of information from the primary auditory cortex to the superior temporal gyrus and inferior frontal gyrus in the MMN context.^[^ ^20^, ^21^ ^]^ Similarly, DCM studies in vision have shown that imprecise target motion increases the gain of superficial pyramidal cells in visual cortex.^[^ ^22^, ^23^ ^]^ Beyond automatic processing, DCM has also been applied to social communication, demonstrating that social demands preferentially modulate backward connections from the medial prefrontal cortex (MPFC) rather than forward connections from the superior occipital gyrus (SOG) and medial temporal gyrus (MTG) to the MPFC.^[^ ^24^ ^]^ Additionally, studies combining DCM with transfer entropy have provided further insights into predictive coding in aging, multisensory integration, and conscious processing, showing that older adults rely more on cross‐modal predictive neural templates.^[^ ^25^ ^]^

Despite the substantial focus on predictive coding, long‐term memory and predictive processes for temporally unfolding information have historically received little attention. This was rather surprising, given that most stimuli humans encounter are inherently structured over time. Thus, as this realization emerged, the topic has, in recent years, attracted significant interest. For example, Albouy and colleagues^[^ ^26^ ^]^ investigated the neural mechanisms underlying memory retention for temporal sequences, demonstrating that theta oscillations in the dorsal stream predict auditory working memory performance. Similarly, Quiroga‐Martinez and colleagues^[^ ^27^ ^]^ showed that predictive processes and the manipulation of temporally structured sound information can be directly decoded from non‐invasive neurophysiological data, revealing a widespread network encompassing the auditory cortices and medial temporal lobe.

Along this line, we conducted a series of studies using diverse tools to estimate dynamic functional connectivity during the long‐term encoding and recognition of temporal sequences. This revealed a large network of brain regions involved in processing both memorized and novel musical sounds, extending from the auditory cortex to the medial cingulate, inferior temporal cortex, insula, frontal operculum, and hippocampus, and showing precise dynamical changes in response to each sound of the sequences.^[^ ^28^, ^29^, ^30^ ^]^ Moreover, we discovered that the activity of this brain network was modulated by musical complexity^[^ ^31^ ^]^ and individual cognitive differences.^[^ ^32^ ^]^ In a subsequent study, following common practice in predictive coding research, we also employed DCM to examine directional functional connectivity between six predefined large‐scale regions of interest (ROIs) during long‐term recognition and prediction of musical sequences. This analysis provided the strongest model evidence for a network in which information flowed from auditory cortices to higher‐order regions such as the hippocampus and ventromedial prefrontal cortex (vmPFC) in both forward and backward directions.^[^ ^33^ ^]^ However, despite being informative, DCM typically involves comparing a hypothesized model against only a limited set of alternatives. Thus, to complement this approach, we recently extended our investigation using a novel multivariate framework, Directed Multiplex Visibility Graph Irreversibility (DiMViGI), which systematically assessed all possible ROI configurations to identify those exchanging the highest amount of information. This revealed distinct peak interactions between the auditory cortex, hippocampus, and vmPFC within each hemisphere, indicating a functionally segregated yet interactive network supporting long‐term recognition of temporal information.^[^ ^34^ ^]^

Although these studies significantly advanced our understanding of auditory predictive coding, they remained constrained to a predefined set of ROIs, fundamentally limiting a comprehensive characterization of whole‐brain connectivity and raising a fundamental question: How are auditory cortices embedded within large‐scale brain networks, potentially engaging in multiple concurrent computations to support predictive and memory processes?

To address this, we draw on previous studies employing linear decomposition techniques to disentangle overlapping brain processes. These methods have been successfully applied in fMRI research, particularly through Principal Component Analysis (PCA) and Independent Component Analysis (ICA)^[^ ^35^, ^36^, ^37^, ^38^, ^39^ ^]^ (for a review, see^[^ ^40^ ^]^). However, their application to predictive and memory processing has been limited, likely due to the low temporal resolution of fMRI,^[^ ^41^ ^]^ which does not allow the computation of reliable covariance matrices for rapid events. By contrast, neurophysiological recordings (EEG/MEG) offer superior temporal resolution, enabling the investigation of rapid covariance patterns over brief time windows, as required for predictive coding and memory studies. Nonetheless, traditional EEG/MEG analyses have primarily applied PCA at the scalp level,^[^ ^42^, ^43^, ^44^, ^45^, ^46^ ^]^ limiting insights into source‐level networks (for a review, see^[^ ^47^ ^]^). One exception is a study that used ICA to derive resting‐state brain networks from MEG data,^[^ ^48^ ^]^ revealing configurations similar to those observed in fMRI. However, this study focused solely on canonical frequency bands in the resting state, offering little insight into event‐related network dynamics and their variance contributions.

To overcome these limitations, we recently developed Network Estimation via Source Separation (NESS), a multivariate framework that applies linear decomposition algorithms to estimate brain networks from source‐reconstructed MEG data.^[^ ^49^ ^]^ Unlike previous methods, NESS directly identifies simultaneous brain networks, providing their explained variance, fine‐grained spatial configurations for physiological interpretation, and activation time series for further analysis. Its frequency‐resolved variant, FREQ‐NESS,^[^ ^49^ ^]^ was recently shown to successfully distinguish concurrent, frequency‐specific networks during resting state as well as their reorganisation in response to auditory stimuli.

In this work, we introduce BROAD‐NESS (BROADband brain Network Estimation via Source Separation), a novel analytical pipeline designed to estimate and characterize broadband whole‐brain networks from source‐reconstructed MEG data. At its core, BROAD‐NESS uses PCA to decompose neurophysiological activity into temporally orthogonal brain networks. However, the pipeline goes beyond standard PCA by integrating additional analytical layers specifically developed or adapted to examine both the spatial and temporal properties of the resulting networks. These include phase space embedding and recurrence quantification analysis (RQA) of network time series to assess the dynamical structure of network trajectories, as well as spatial gradients embedding and clustering of brain voxels to identify anatomically coherent patterns of network participation. Inspired by previous works which employed similar techniques in other domains (e.g., resting‐state fMRI spatial gradients),^[^ ^50^, ^51^, ^52^ ^]^ we adapted and combined these analytical tools within BROAD‐NESS to create a novel, streamlined, data‐driven, and physiologically interpretable pipeline tailored to the analysis of event‐related brain networks in MEG. Thus, the aim of this study is twofold. Methodologically, we present and validate BROAD‐NESS as a flexible and interpretable framework for investigating both the spatial organization and temporal dynamics of whole‐brain networks in neurophysiology. Conceptually, we demonstrate the immediate utility of BROAD‐NESS by applying it to a robust auditory memory recognition paradigm, yielding novel insights into how large‐scale brain networks support predictive coding and long‐term memory for temporally structured sounds.

## 2 Results

### 2.1 Overview of the Experimental Design and MEG Source Reconstruction

**Figure 1:**
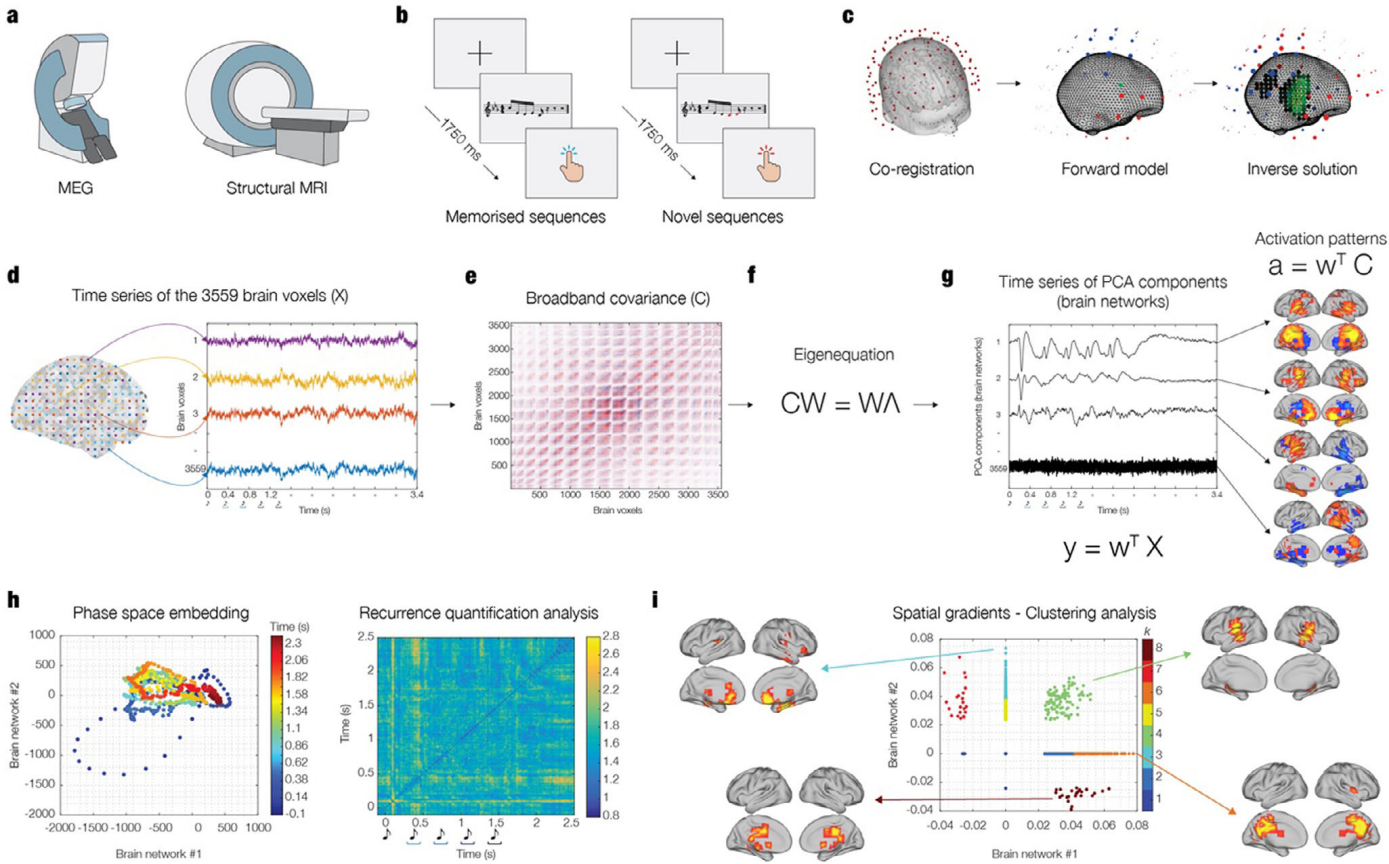
BROADband network estimation via source separation (BROAD‐NESS). The figure provides an overview of the BROAD‐NESS methodology. a) MEG was used to collect neurophysiological data while participants performed an auditory old/new recognition task. b) Five‐tone auditory sequences, presented in random order, were classified by participants as either “old” (memorised sequences, M) or “new” (novel sequences, N) using button presses. c) Co‐registration was performed between MEG data and individual MRI anatomical scans. To reconstruct the neural sources which generated the signal recorded by the MEG, a single shell forward model was used. The inverse solution was estimated through beamforming. d) The source reconstruction yielded time series data for 3,559 brain voxels based on an 8‐mm grid brain parcellation. e) The covariance matrix (C) was computed from the broadband voxel data matrix. f) Principal Component Analysis (PCA) was computed by solving the eigenequation CW = WΛ for the eigenvectors (W) to find the weighted combinations of brain voxels to orthogonal components, which reflected brain networks; the associated eigenvalues (Λ) express the amount of variance explained by each network component. g) Network activation time series (y) were derived by applying the spatial filters (w) to the voxel data matrix (X). The same filters were applied to the covariance matrix (C) to compute the spatial activation patterns (a), representing the components' projections in voxel space. h) Temporal embedding and recurrence quantification analysis (RQA) were applied to the network time series to investigate their dynamic temporal properties. Phase space plots (left) and recurrence matrices (right) captured differences in trajectory recurrency and complexity across experimental conditions, enabling quantitative analysis of the dynamics of embedded networks and their link to behavioural performance. i) To examine the spatial organisation of voxel contributions, we performed a spatial gradient embedding and clustering analysis based on the spatial activation patterns of the two main BROAD‐NESS‐derived brain networks. The contributions of individual voxels were embedded in a 2D space defined by the first two principal components, visualising their joint involvement across networks. An unsupervised clustering procedure applied to this space revealed anatomically interpretable voxel groups, including clusters engaged in both networks, selectively involved in one, or showing opposing contributions. These clusters were then mapped back onto brain templates, demonstrating their spatial coherence and relevance to large‐scale brain organisation.

Eighty‐three participants engaged in an old/new auditory recognition task during magnetoencephalography (MEG) recordings (**Figure** **1**a,b). Following the learning of a brief musical piece (see Figure S1, Supporting Information), participants were presented with 135 five‐tone musical sequences, each lasting 1750 ms (each sequence comprised five musical notes lasting 350 ms each). Participants were asked to indicate whether each sequence belonged to the original music (‘memorized’ sequence, denoted as M, old) or represented a varied sequence (‘novel’ sequence, denoted as N, new) (Figure 1a). Among the presented sequences, 27 were directly extracted from the original musical piece, while 108 were variations. These variations were categorized based on the number of musical tones altered following the first tone (NT1), second tone (NT2), third tone (NT3), or fourth tone (NT4) (see Figure S2, Supporting Information).

After performing the pre‐processing of the MEG data (see Experimental Section for details), we computed source reconstruction. We employed a single‐shell forward model alongside a beamforming approach as the inverse solution, using an 8‐mm grid that corresponded to 3559 brain voxels (Figure 1c,d). This procedure produced a time series for each reconstructed brain voxel returning a matrix of 3559 brain voxels x time‐points, independently for each participant and experimental condition.

We conducted statistical analyses on the behavioral data from the MEG task (**Table** **1**). Two independent Kruskal–Wallis H tests were performed to examine whether the five auditory sequence categories (M, NT1, NT2, NT3, NT4) differed in terms of response accuracy and reaction times. The Kruskal–Wallis test for response accuracy revealed a significant effect of sequence category (H(4) = 36.38, *p* <.001), indicating differences in correct recognition rates. Post hoc comparisons with Tukey–Kramer correction showed that NT4 trials were recognized significantly less accurately than M (*p* = 0.001), NT1 (*p* = 0.001), NT2 (*p* = 0.0003), and NT3 trials (*p* < 0.0001). Similarly, the Kruskal–Wallis test for reaction times was significant (H(4) = 22.53, *p* = 0.0002), indicating that response speed differed across conditions. Post hoc comparisons revealed that NT4 trials elicited significantly longer reaction times compared to M (*p* = 0.0016), NT1 (*p* = 0.0013), NT2 (*p* = 0.0054), and NT3 trials (*p* = 0.0008).

**Table 1:** Behavioural results for the old/new paradigm recognition task.

Behavioral variables	M	NT1	NT2	NT3	NT4
Correct recognition	22.33 ± 5.30	22.36 ± 4.27	21.58 ± 5.31	21.66 ± 5.34	17.04 ± 7.12
Reaction times (ms)	2426 ± 226	2407 ± 284	2431 ± 282	2415 ± 272	2578 ± 259

Mean (± standard deviation) number of correctly recognised trials and reaction times (in ms) across participants for each of the five experimental conditions: previously memorised (M) and novel types NT1, NT2, NT3, and NT4. Each participant was presented with 27 trials per condition.

### 2.2 PCA, Monte Carlo Simulations (MCS) and Statistics

PCA was conducted on the reconstructed time series of the 3559 brain voxels, averaged across participants and conditions, to identify simultaneously operating broadband brain networks (Figure 1e–g). To assess significant Principal Components (PCs), a Monte Carlo Simulation (MCS) approach was used by performing PCA on data randomized across the temporal dimension for 100 permutations. Significant principal components from the original data were defined as those explaining more variance than the maximum variance accounted for by the first component across the 100 permutations of the randomized data (see Experimental Section for details). Remarkably, the variance explained by the principal components in the randomized data remained rather consistent across all permutations, suggesting that a conventional set of 100 permutations may not be necessary since a reduced number of permutations (e.g., 50 or even as few as 10) would indeed yield equivalent results.

This procedure identified six significant PCs, which accounted for the following percentages of variance: 71.75%, 16.32%, 4.16%, 1.30%, 0.99%, 0.92%. Each component was interpreted as a distinct brain network based on its spatial activation pattern, which was obtained by multiplying the component's eigenvector by the covariance matrix of the data. Unlike FREQ‐NESS, which was based on GED,^[^ ^53^, ^54^, ^55^, ^56^, ^57^, ^58^ ^]^ the weights and spatial activation patterns produced by PCA were equivalent in demonstrating the relative contribution of each brain voxel to the network.

**Figure 2:**
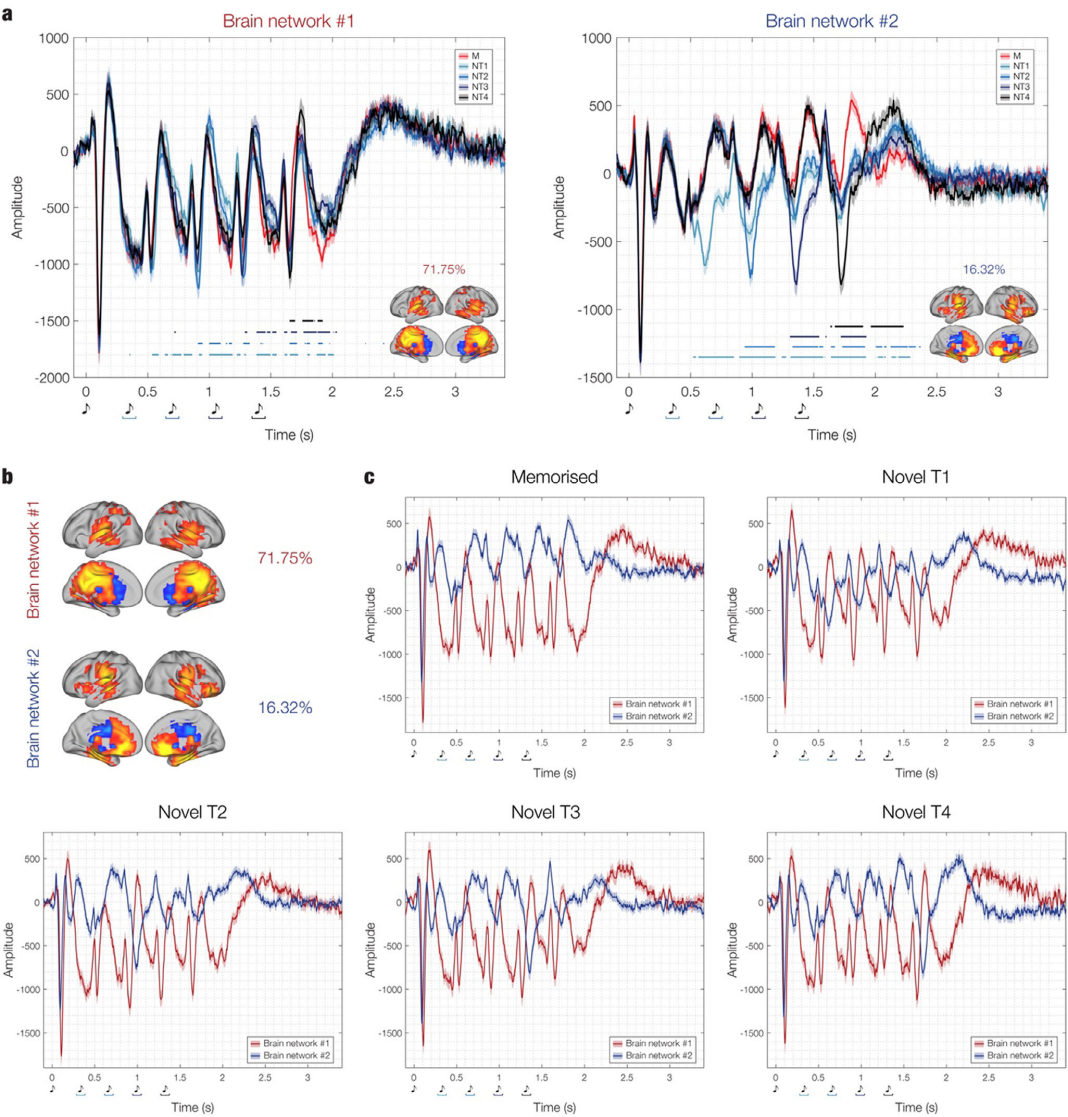
Time series and spatial activation patterns of the main brain networks. The figure illustrates the time series and spatial activation patterns of the two brain networks that explained the highest variance (71.75% and 16.32%, respectively). These networks were estimated using PCA within the BROAD‐NESS framework, computed on the data averaged across conditions and participants. a) Independent time series for each participant, brain network, and experimental condition were generated using PCA‐derived weights from the averaged data. The individual time series were then averaged across participants, as shown in the plots. Shaded areas represent standard errors. The brain templates illustrate the spatial extent of the networks, with yellow voxels contributing the most and light blue voxels contributing the least to the time series. Only voxels with values exceeding the mean by more than one standard deviation in absolute terms are depicted. Blue‐black lines indicate the temporal extent of the significant differences between M versus each category of N (i.e. M versus NT1, M versus NT2, M versus NT3, M versus NT4), computed using two‐sided *t*‐tests corrected for multiple comparisons via False Discovery Rate (FDR). Different shades of blue‐black represent specific M versus N comparisons. b) Focus on the spatial activation patterns of the brain networks. c) Focus on the time series of the brain networks, depicted independently for each experimental condition to emphasize similarities and differences between the two brain networks time series. Musical tone sketches represent the onset of each tone in the sequences, while the coloured graphs indicate the tone where the variation was introduced in the different N conditions.

As illustrated in **Figure** **2**, the first identified brain network comprised the auditory cortices and the medial cingulate gyrus, while the second one included the auditory cortices, anterior cingulate gyrus, ventromedial prefrontal cortex, hippocampal regions, and insula. The remaining four networks were characterized by similar spatial maps; however, they were deemed less relevant as they explained considerably less variance (Figure S3, Supporting Information).

The weights were subsequently multiplied by the original data to compute the time series for each brain network. It is important to note that while the weights were computed using data averaged across participants and conditions, the computation of the brain network time series was performed independently for each participant and experimental condition. This was necessary to allow statistical comparisons of the brain networks time series across experimental conditions. Specifically, statistical analyses were conducted by contrasting the time series of the memorized versus varied musical sequences, consistent with our prior study focusing on specific brain ROIs. Here, a two‐sided t‐test was performed for each time point and each combination of memorised versus novel sequences (i.e., M versus NT1, M versus NT2, M versus NT3, M versus NT4). Multiple comparisons were corrected using False Discovery Rate (FDR), giving rise to the following FDR thresholds for the *p*‐values: 0.0210 (brain network #1, M vs NT1); 0.0105 (brain network #1, M vs NT2); 0.0118 (brain network #1, M vs NT3); 0.0043 (brain network #1, M vs NT4); 0.0319 (brain network #1, M vs NT1); 0.0241 (brain network #1, M vs NT2); 0.0109 (brain network #1, M versus NT3); 0.0107 (brain network #1, M versus NT4). This analysis revealed several significant time points where the memorized sequences differed significantly from the novel ones (Figure 2). Detailed statistical results are summarized in Table S1 (Supporting Information).

### 2.3 Phase Space and Recurrence Quantification Analysis (RQA)

**Figure 3:**
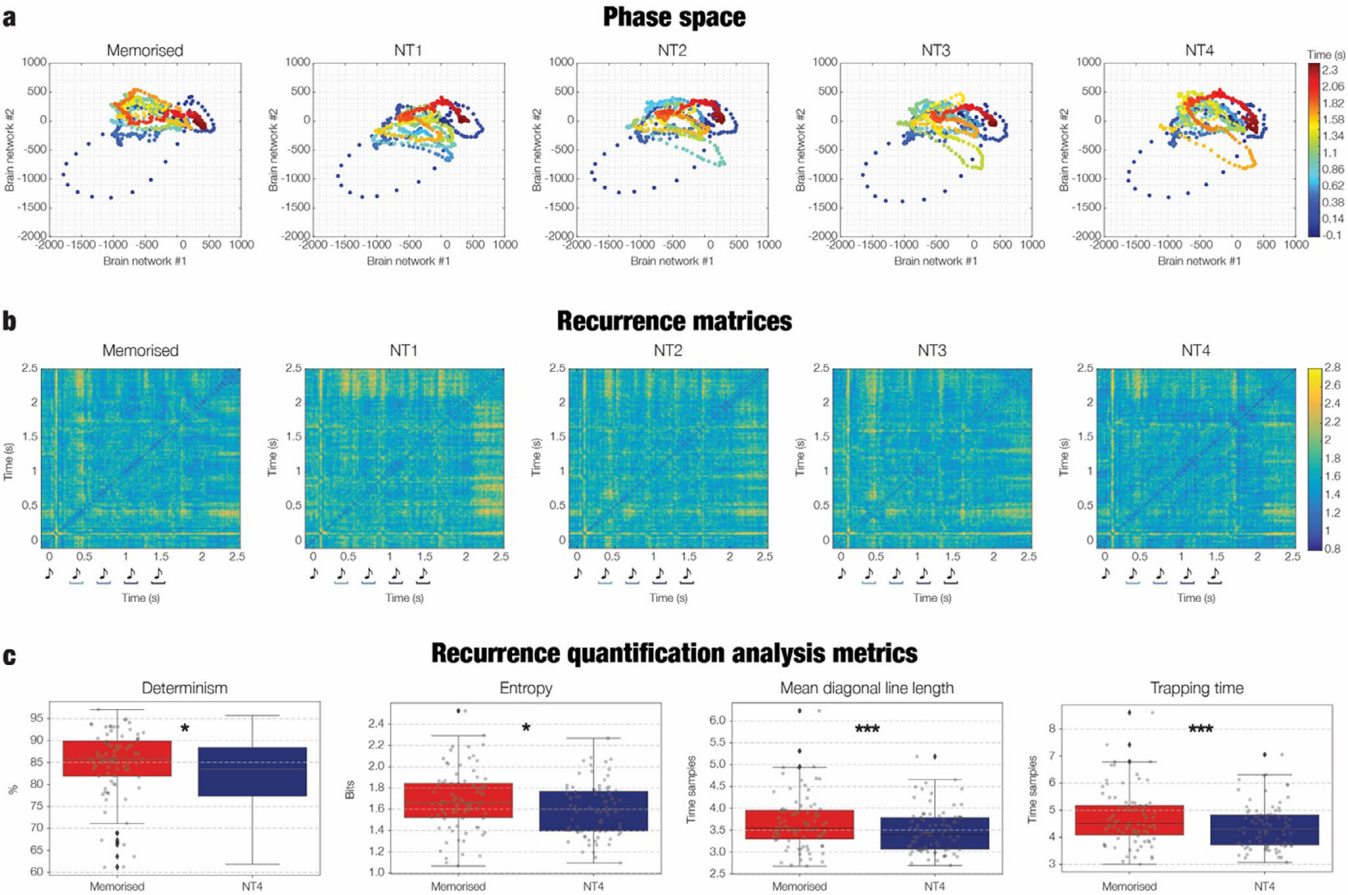
Phase space embedding and recurrence analysis of BROAD‐NESS‐derived brain networks dynamics across conditions. a) Phase space trajectories averaged across participants (n = 83) for each experimental condition, obtained by embedding the time series of the two primary BROAD‐NESS networks into a 2D state space. Each dot represents the state of the brain at a given time point, color‐coded by time (in s). Compared to the novel tone conditions (NT1–NT4), the memorized condition (M) shows more compact and coherent trajectories in phase space. b) Recurrence matrices computed from the phase space trajectories shown in a) and averaged across participants (n = 83), depicting pairwise Euclidean distances between time points. Warmer colors indicate larger distances (i.e., less similar brain states), while cooler colors indicate greater similarity and temporal recurrence. c) Boxplots of four recurrence quantification analysis (RQA) metrics: determinism (DET), entropy (ENTR), mean diagonal line length (L), and trapping time (TT). They illustrate the statistical comparison between the M and NT4 conditions. These metrics quantify the predictability, complexity, and temporal persistence of network state trajectories. All four metrics were significantly higher for the M condition (n = 83 participants), suggesting more stable and structured dynamics during successful memory‐based recognition. Significance levels: * *p* < 0.05, ** *p* < 0.01, *** *p* < 0.001; all p‐values were significant after false discovery rate (FDR) correction. Musical tone sketches represent the onset of each tone in the sequences, while the colored graphs indicate the tone where the variation was introduced in the different N conditions.

To further characterize the temporal organization of the two main brain networks identified by BROAD‐NESS, we analyzed their dynamics in a joint 2D phase space and computed recurrence‐based metrics. Visual inspection of the phase space trajectories (**Figure** **3**a) revealed coherent trajectories for M, with tighter, more structured patterns compared to the novel tone conditions. Recurrence plots constructed from these trajectories (Figure 3b) confirmed this impression, showing a denser and more organized structure for M than for the novel conditions, particularly NT4. Quantitative statistical comparisons (Figure 3c) of recurrence metrics showed significant differences between M and NT4 for four of the eight extracted measures (FDR thresholds for *p* = 0.0233). Specifically, the memorized condition exhibited significantly higher mean diagonal line length (*p* = 0.0008), determinism (*p* = 0.0233), entropy (*p* = 0.0023), and trapping time (*p* = 0.0008), indicating that temporal trajectories in M were more structured, predictable, and persistent. No other comparisons survived the correction for multiple comparisons. These results suggest that during memory recognition, the brain settles into more stable and recurrent network dynamics compared to conditions where the final tone deviates from the memorized sequence.

### 2.4 Correlation of Recurrence Metrics with Behavioral Performance

To test whether recurrence properties of network dynamics were related to behavioral outcomes, we computed Spearman's rank‐order correlations between each participant's average recurrence metrics and their overall accuracy and reaction times (RTs) in the memory task. As shown in **Table** **2**, seven out of eight recurrence measures showed statistically significant correlations after FDR correction (FDR thresholds for *p* = 0.0113). Specifically, higher accuracy was associated with more structured, rich and persistent network trajectories, reflected in positive correlations with metrics such as mean diagonal line length, determinism, entropy, trapping time, laminarity and maximum vertical line length. Similarly, faster RTs showed the same pattern, as indicated by negative correlations with the same metrics. Conversely, divergence, reflecting sensitivity to initial conditions and neural instability, was negatively associated with both accuracy and RTs, pointing to the detrimental role of disordered or chaotic network embedded dynamics for the performance. Altogether, these findings suggest that individuals with more structured and recurrent brain networks dynamics tended to perform more accurately and respond more quickly in the memory task.

**Table 2:** Spearman's rank‐order correlations between average recurrence metrics and behavioural performance.

Spearman's correlation	L	DET	ENTR	TT	LAM	Vmax	DIV
Accuracy							
*r*	0.3971	0.4017	0.4015	0.3923	0.3874	0.3972	−0.4567
*p*‐value	2.01e‐04	1.67e‐04	1.69e‐04	2.45e‐04	2.97e‐04	2.01e‐04	1.43e‐05
RTs							
*r*	−0.2960	−0.2924	−0.3026	−0.2991	−0.2775	−0.2919	0.3045
*p*‐value	0.0068	0.0075	0.0056	0.0062	0.0113	0.074	0.0053

Correlation coefficients (*r*) and associated *p*‐values are shown for both accuracy and reaction time (RT) measures, averaged across task conditions. Seven out of eight recurrence metrics were significantly correlated with accuracy after FDR correction (threshold *p* = 0.0178), indicating that higher behavioural accuracy was associated with more structured and recurrent brain dynamics. RTs correlations suggested that more stable dynamics were associated with faster responses. Metrics included: L (mean diagonal line length), DET (determinism), ENTR (entropy), TT (trapping time), LAM (laminarity), Vmax (maximum vertical line length), and DIV (divergence; inverse of the longest diagonal line).

### 2.5 Spatial Gradients Embedding of BROAD‐NESS‐Derived Network Topographies

**Figure 4:**
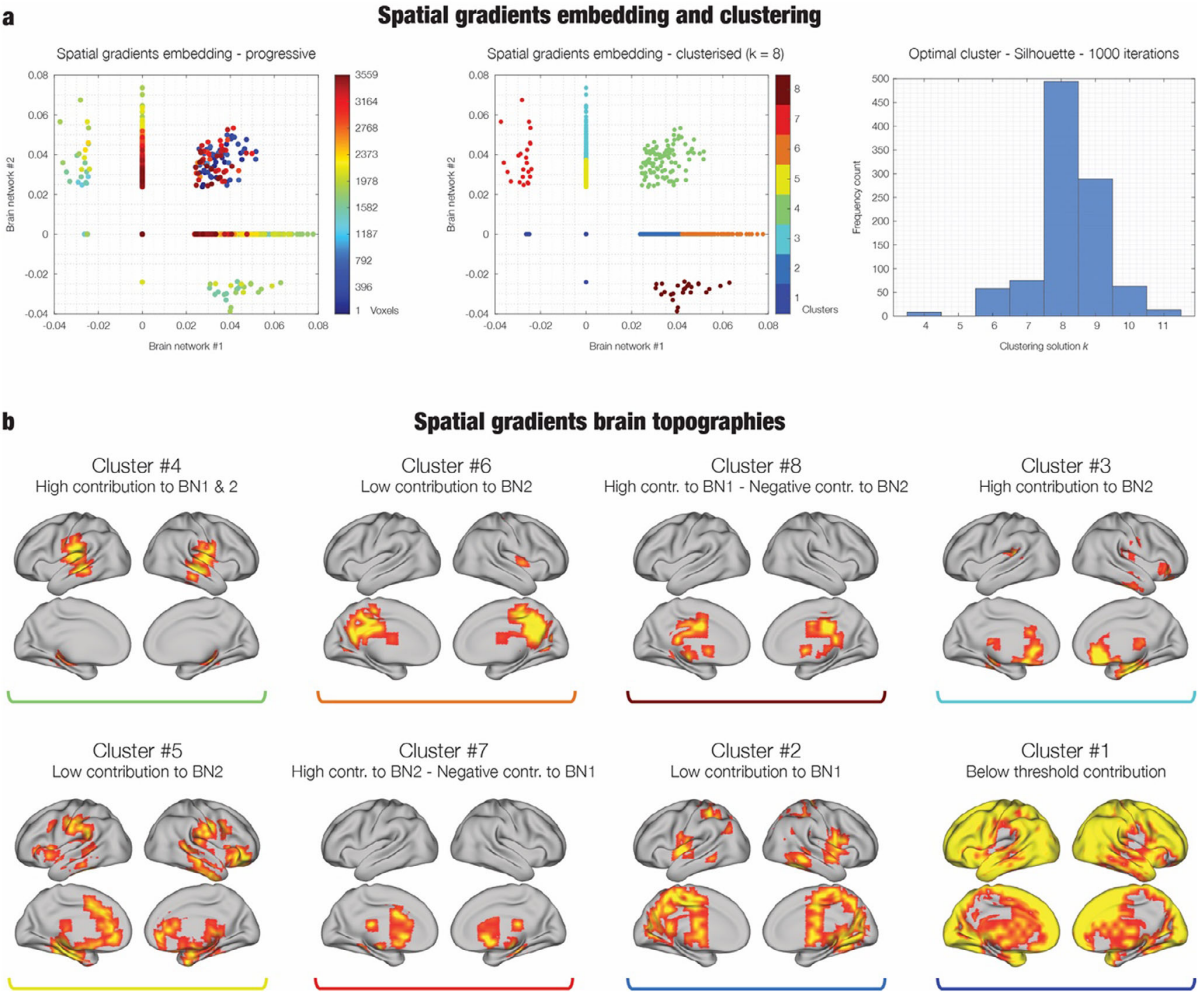
Spatial gradients embedding and voxel clustering analysis. This figure illustrates the analysis of voxel‐wise spatial activation patterns derived from the two main BROAD‐NESS brain networks. a) 2D scatter plot showing the distribution of all brain voxels according to their spatial activation values in the first two principal components (PC1 and PC2), revealing the underlying gradient structure of network involvement (left). Same 2D embedding with voxels colored according to the eight clusters identified by the optimal clustering solution, highlighting distinct patterns of co‐involvement across the two networks (right). b) Histogram of silhouette coefficients across 1000 clustering iterations for each tested solution (k = 2 to 20). The peak at k = 8 confirms the optimal number of spatial clusters, based on maximum silhouette consistency. c) Brain template projections of the eight clusters from the optimal solution. These maps illustrate the spatial distribution of voxel groups identified in panel a), revealing coherent and anatomically interpretable clusters, including shared, selective, and opposing contributors to the two BROAD‐NESS networks.

To further characterize the spatial organization of the BROAD‐NESS‐derived networks, we applied a data‐driven gradient and clustering approach to the voxel‐wise activation maps of the first two principal components (PC#1 and PC#2). These components represent the two most dominant whole‐brain networks involved in the auditory recognition task. Each voxel was embedded in a 2D space according to its contribution to PC#1 and PC#2 (thresholded at mean ± 1 standard deviation), enabling a compact spatial representation of network co‐involvement across the brain. Clustering analysis using k‐means (performed on z‐scored values and repeated 100 times per *k*, ranging from two to 20) revealed that an eight‐cluster solution provided the most robust and reproducible partitioning of voxels in this space, as evaluated using silhouette coefficients. To ensure the reliability of this clustering solution, we repeated the entire clustering and silhouette evaluation procedure 1000 times and consistently found that *k* = 8 yielded the highest silhouette scores across most repetitions. This optimal solution is visualized in **Figure** **4**, which shows the identified clusters projected back onto anatomical brain templates, while detailed information on the brain voxels forming these eight clusters is reported in Table S2 (Supporting Information). For full transparency, Figure S4 (Supporting Information) displays clustering results across the full range of *k* values, highlighting the robustness of the solution and the consistency of the results across adjacent clustering levels. The eight‐cluster structure provided a clear and interpretable mapping of how different voxel populations contributed to the two dominant networks. This analysis confirmed the presence of voxels strongly engaged in both networks, particularly within the bilateral primary and secondary auditory cortices. We also observed voxels selectively contributing to only one network: the medial and posterior cingulate gyrus for Network #1, and the anterior cingulate and right hippocampus for Network #2. In addition, some voxels exhibited opposing contributions across the two networks. For example, voxels in the medial cingulate gyrus contributed positively to Network #1 but negatively to Network #2, while the reverse was observed in the anterior cingulate. Finally, the analysis revealed spatially coherent clusters of voxels with minimal or no contribution to either network, likely representing non‐task‐specific background activity. These regions encompassed much of the occipital lobe, unimodal sensory areas (e.g., visual and sensorimotor cortices), and frontal regions not implicated in the current task.

### 2.6 Behavioral Measures and Brain Networks Activity

**Figure 5:**
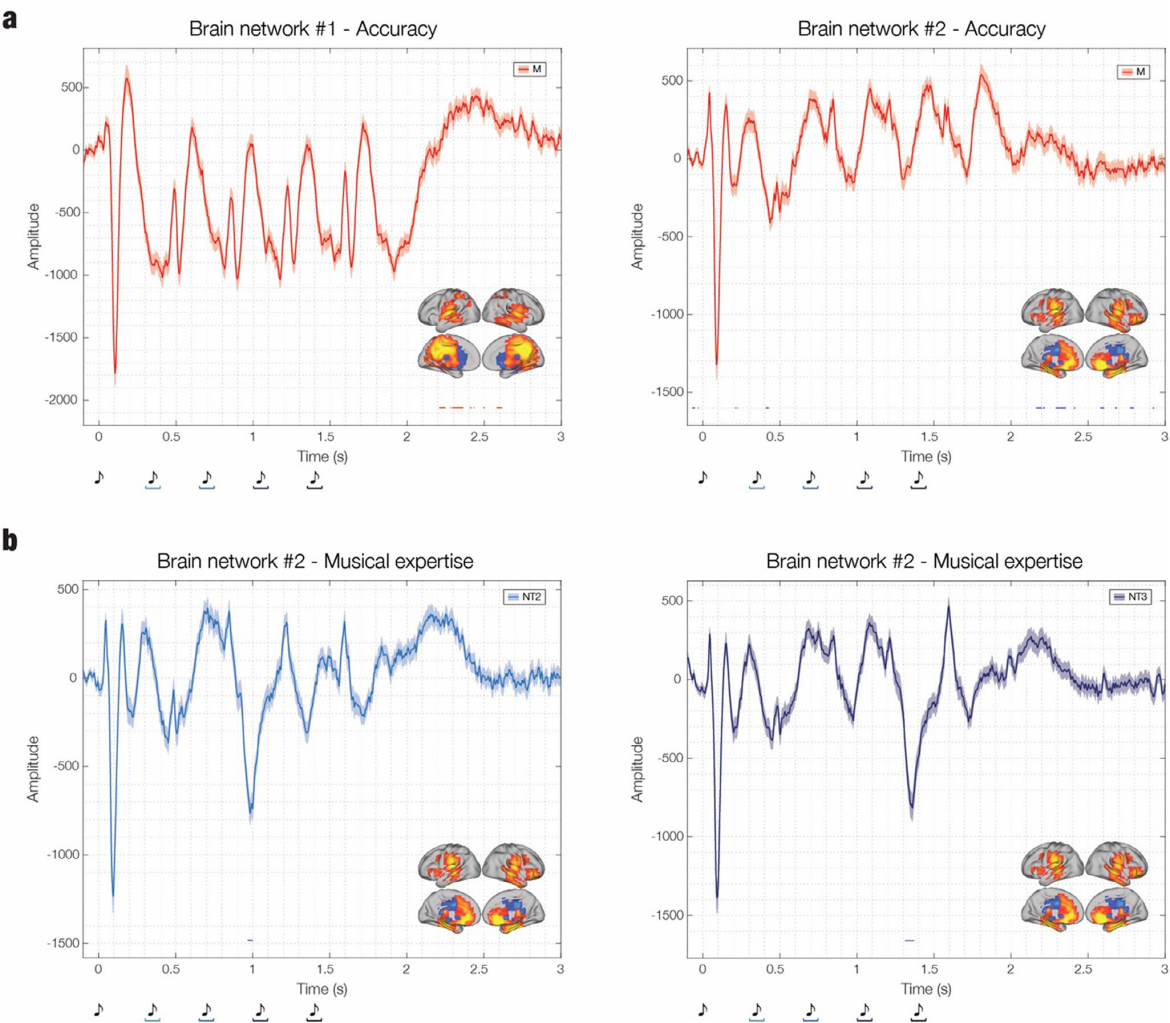
Relationship between BROAD‐NESS‐derived brain networks activity and behavioural variables. a) Time series of the two main BROAD‐NESS networks during the memorized condition (M), averaged across participants (n = 83). For each network, the signal is plotted with shaded areas indicating standard errors. Red and blue horizontal lines indicate time intervals where a significant correlation was found between network amplitude and accuracy (false discovery rate [FDR]‐corrected). Both network #1 (left) and #2 (right) showed stronger correlations in the later part of the trial, indexing that the higher the accuracy the higher the neural activity for network #1 and the lower for network #2. Brain templates display spatial maps of the respective networks, with warmer colors indicating stronger spatial activation patterns. b) Time series of brain network #2 in the NT2 and NT3 conditions, averaged across participants (n = 83). Blue horizontal lines indicate time intervals where a significant negative correlation was found between musical expertise and network amplitude (FDR‐corrected). Effects were more prominent following deviant tones in positions three (NT2) and four (NT3), suggesting that higher musical expertise was associated with enhanced conscious prediction errors. Brain templates show the spatial distribution of brain network #2.

To further examine the relationship between behavioral performance, individual differences, and the brain networks identified in the present study, we conducted correlation analyses between the time series of the two networks extracted via the BROAD‐NESS analytical pipeline and three behavioral measures: (i) accuracy on the memory task during MEG recording, (ii) RTs, and (iii) musical expertise. Multiple comparisons were controlled using the FDR method. The results are illustrated in **Figure** **5**, with detailed statistical information (including specific time points and correlation coefficients) presented in Table S3 (Supporting Information). The time series following presentation of the memorized musical sequence showed significant correlations with accuracy in both brain networks (FDR thresholds for *p* = 0.0029 for brain network #1; *p* = 0.0051 for brain network #2). These findings suggest that neural activity in this time window may reflect cognitive processes which are particularly relevant for decision‐making related to recognition of the memorized sequences, rather than the varied ones. In contrast, correlations with RTs were less robust but involved a broader range of experimental conditions, revealing weaker yet statistically significant associations between network activity and response speed (FDR thresholds for *p* = 3.03e‐04 for brain network #1, NT1; *p* = 0.0041 for brain network #2, M; *p* = 0.0018 for brain network #2, NT3; *p* = 0.001 for brain network #2, NT4). Finally, musical expertise was significantly associated with the prediction error responses in brain network #2 when a deviant sound was introduced at position three (FDR thresholds for *p* = 6.67e‐04) or four (*p* = 9.87e‐04) within the sequence, indicating a modulatory effect of expertise on processing unexpected auditory events.

### 2.7 ICA Decomposition and Comparison with PCA within BROAD‐NESS

**Figure 6:**
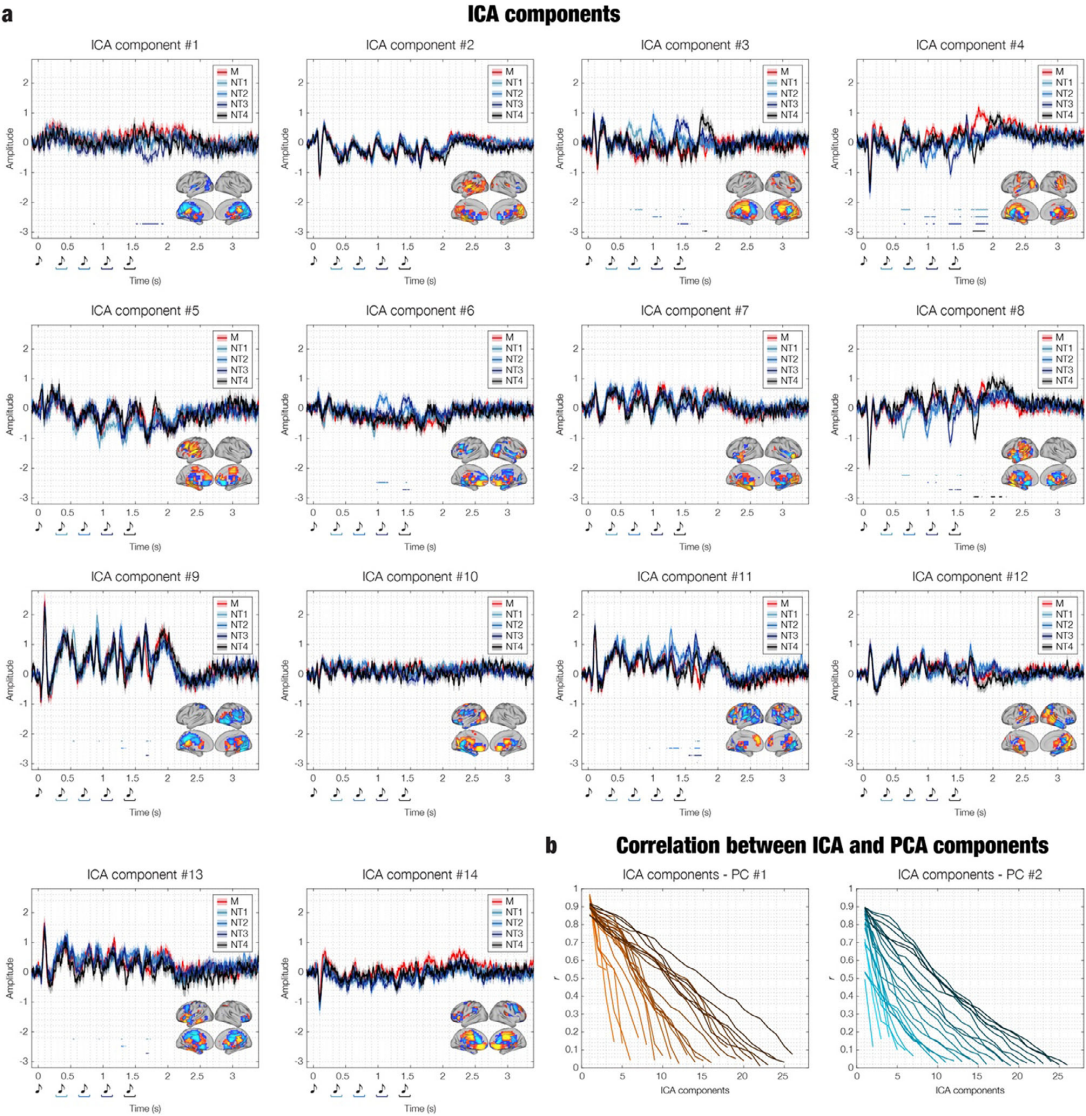
Brain networks obtained through Independent Component Analysis (ICA) and correspondence with BROAD‐NESS‐PCA‐derived brain networks. a) Time series and spatial maps in brain templates (threshold for visualization = mean plus one standard deviation) of the 14 independent components extracted via ICA on source‐reconstructed MEG data averaged across participants (n = 83). Each line represents the mean time series per condition (M = memorized, NT1–NT4 = novel tone conditions), with shaded areas indicating standard errors. Horizontal colored lines denote time points at which statistically significant differences (two‐sided t‐tests, false discovery rate [FDR]‐corrected, *p* < 0.05) were observed between pairs of conditions (M vs NT1, M vs NT2, M vs NT3, M vs NT4). Components varied in temporal stability and condition sensitivity, with some (e.g., ICA #3, #4, #6, #8) reflecting plausible task‐related networks involving cingulate cortex, auditory cortex, hippocampus, and prefrontal regions. However, ICA‐derived components were generally less interpretable and exhibited greater spatial dispersion compared to PCA‐BROAD‐NESS results. b) Pearson's correlation between the time series of the first two BROAD‐NESS principal components (PC#1 and PC#2) and all ICA components across multiple ICA decompositions, ranging from 2 to 26 components (with 14 and 25 components corresponding to 95% and 99% of the PCA‐explained variance, respectively). In all cases, a linear decrease in correlation was observed across ICA components, regardless of the total number extracted. This pattern indicates a systematic correspondence between early ICA components and the dominant PCA‐derived networks, while also highlighting a limitation of ICA: it does not offer a principled nor easily interpretable criterion for selecting either the most meaningful components or the optimal number of components to extract. Musical tone sketches represent the onset of each tone in the sequences, while the colored graphs indicate the tone where the variation was introduced in the different N conditions.

To further examine the structure and functional validity of the networks identified via BROAD‐NESS‐based PCA, we performed an ICA decomposition on the same source‐reconstructed MEG data. ICA was first computed by extracting 14 components, corresponding to the number of PCA components required to capture 95% of the total variance. Statistical comparisons of the ICA‐derived time series across conditions (M vs NT1, NT2, NT3, NT4) revealed several significant time points. However, these effects were confined to a smaller subset of components and were overall less consistent and extended in time compared to the results from PCA BROAD‐NESS. Some ICA components did capture meaningful condition‐specific differences and exhibited spatial activation patterns resembling those of PCA‐derived brain networks, though the maps appeared more scattered and topographically less coherent (**Figure** **6**). Full statistical results for this 14‐component solution are reported in Table S4 (Supporting Information).

In parallel, we also examined a 2‐component ICA solution to match the two main networks identified via PCA. This decomposition showed clear temporal and spatial similarity with the first BROAD‐NESS network, and to a lesser extent with the second one. However, in this lower‐dimensional ICA solution, differences between experimental conditions were not pronounced, and the resulting components lacked the distinct functional specificity observed in PCA (Figure S5, Supporting Information). Statistical comparisons for the two‐component ICA decomposition are reported in Table S4 (Supporting Information).

To quantify the correspondence between ICA and BROAD‐NESS, we computed Pearson correlation coefficients between each ICA component time series and the time series of the two main PCA‐derived networks. Correlations were computed independently for each experimental condition and then averaged across conditions. As shown in Figure 6, the strongest correlations were typically found for a small subset of ICA components that qualitatively resembled the PCA time series. To systematically assess this relationship, we repeated the analysis across multiple ICA decompositions, varying the number of extracted components from two to 26. For each ICA solution, we computed the correlation between all ICA components and the PCA‐derived network of interest. This revealed a consistent pattern: within each ICA solution, correlation values tended to decrease linearly from the most to the least similar component, regardless of the total number of components extracted. This indicates that increasing ICA dimensionality did not qualitatively alter the structure of similarity with the PCA networks but rather extended the correlation decay curve in a predictable, ordered fashion.

### 2.8 Data Randomization and Brain Networks

In addition to the MCS approach, we aimed to systematically assess how the sensitivity of BROAD‐NESS was influenced by the temporal and spatial dimensions of latent brain networks. To achieve this, we independently disrupted the spatial and temporal organization of the data matrix (brain voxels x time points) by implementing two types of randomization.

The randomization strategies were designed as follows: (i) space randomization, where the voxel indices were shuffled row‐wise; and (ii) time randomization, where the time indices were shuffled independently for each brain voxel. As in the case of the Monte Carlo simulations, to evaluate the stability and robustness of the procedure, each randomization strategy was executed 100 times.

For the first randomization (space randomization), we found that the variance explained by the principal components and their associated time series remained identical to those of the original data (Figure S6, Supporting Information). However, their spatial activation patterns exhibited a completely disrupted structure, indicating that this randomization did not affect the PCA computation but resulted in non‐meaningful spatial extents of the brain networks. In contrast, the second randomization (time randomization, which was also used within the MCS approach detailed above) disrupted both the spatial and temporal domains, leading to a significantly reduced variance explained by the main components (Figure S6, Supporting Information). In this case, the combination of disrupted spatial activation patterns and time series displaying no differences between experimental conditions made the estimated brain networks not interpretable. The same statistical testing described for the original data was performed for both randomization strategies. The time series and spatial activation patterns obtained from the two randomization strategies are illustrated in Figure S7 (Supporting Information), while additional information and tests on the performance of the permutation algorithms we used are depicted in Figure S8 (Supporting Information). Finally, the complete statistical report is provided in Table S5 (Supporting Information).

### 2.9 Comparing Different PCA Computations

To enhance the robustness of our analysis and elucidate the subtle differences arising from distinct PCA computation approaches to neural data, we conducted additional analyses. A key question relevant to experimental settings is whether it is more advantageous to compute PCA on individual experimental conditions or on aggregated data (e.g., data averaged over conditions).

In this study, we explored both approaches and compared the results, as illustrated in Figure S9 (Supporting Information). Specifically, PCA was first computed on the data averaged across conditions, followed by the computation of time series. We also performed PCA independently for each condition, allowing for a direct comparison of the time series derived from the first PCs and an assessment of the differences. As shown in Figure S9 (Supporting Information), the two computations returned almost identical results, suggesting that the brain networks underlying the different experimental conditions were very similar (yet characterized by different temporal dynamics).

This concept can similarly be applied to the multiple participants involved in the study. PCA can be performed on the average data across participants, followed by the independent reconstruction of time series for each participant using the weights obtained from PCA. Alternatively, PCA can be computed independently for each participant, along with their respective time series, or it can be computed on the concatenated data from all participants and then the time series of each brain network can be reconstructed independently for each participant. We implemented all three approaches and presented a comparison in Figure S10 (Supporting Information), which revealed similar results, with the clearest and most interpretable outcomes obtained from PCA computed on the average data across participants.

### 2.10 Focus on Multiple Comparisons Corrections

To further enhance the robustness of our results and provide additional insights into the contrasts between brain network time series while correcting for multiple comparisons, we computed and compared three additional correction methods specifically for the contrast between M and N1. The approaches, detailed in the methods section, include:(i) Bonferroni correction, (ii) cluster‐based permutation test, and (iii) 1D cluster‐based Monte Carlo Simulation (MCS, α = .05, MCS *p‐*value = 0.001). As illustrated in Figure S11 (Supporting Information) and reported in detail in Table S6 (Supporting Information), the results were highly convergent, confirming the effectiveness and consistency of all three correction methods.

## 3 Discussion

Our study provides new insights into the large‐scale brain networks underlying predictive coding during auditory memory recognition. To this end, we employed BROAD‐NESS, a variant of the recently validated NESS framework,^[^ ^49^ ^]^ which applies PCA for the broadband decomposition of neurophysiological data. This analytical pipeline not only identifies large‐scale orthogonal brain networks but also enables the extraction of additional metrics to characterize their temporal dynamics and spatial distribution, specifically through phase space and spatial gradient embedding analysis. Using this approach, we identified two distinct yet concurrent whole‐brain networks involved in auditory and predictive processing. We revealed how their embedded temporal dynamics, examined through phase space and recurrence quantification analysis (RQA), provided unique insights into participants’ ability to successfully perform the auditory memory task. Furthermore, we demonstrated that individual brain voxels contributed differently to each network, identifying spatially organized clusters that were selectively engaged in either one network, both networks, or that contributed positively to one while negatively to the other. Altogether, these findings go beyond conventional hierarchical models of forward and backward information flow. Moreover, while most prior studies on predictive coding focused on isolated brain regions or small‐scale pairwise connections, our results show that predictive coding mechanisms are embedded within large‐scale, dynamically interacting networks. These networks engage auditory cortices in distinct interactions with multiple higher‐order brain regions and exhibit both fine‐grained spatial gradients and complex temporal organization. Finally, complementary ICA analyses further underscored the advantages of the BROAD‐NESS PCA‐based approach, highlighting its efficiency, robustness, and superior interpretability in extracting meaningful whole‐brain networks. Three key insights emerged from the current study, alongside a number of additional theoretical and methodological considerations.

First, we identified two primary whole‐brain networks that were represented by the first two principal components, together explaining 88.06% of the variance (71.75% and 16.31%, respectively). Both networks prominently involved the auditory cortices but diverged in their broader connectivity and functional implications. The first network, also including the medial cingulate gyrus, captured early auditory processing, such as the N100 elicited by each sound, as well as later, slower ERF components sustaining activity between sounds. This network was particularly prominent and appeared relatively stable regardless of the PCA computation strategy. It emerged clearly whether PCA was computed on averaged data, individual participant data, or concatenated data. Nevertheless, computing PCA on data averaged across participants remained the recommended approach. The second network, in addition to auditory cortices, involved hippocampal regions, the inferior temporal cortex, insula, and prefrontal regions, including the anterior cingulate and ventromedial prefrontal cortices. The time series of this second network indicated a slow positive ERF component for each sound, likely reflecting a process of matching incoming tones to stored memory traces of previous melodies.^[^ ^59^, ^60^ ^]^ Additionally, a more rapid negative peak emerged in response to variations in the sequence, which is consistent with prediction error responses.^[^ ^61^, ^62^ ^]^ These findings align with our previous ROI‐based studies,^[^ ^32^, ^33^ ^]^ which identified hierarchical interactions between a restricted set of brain regions including auditory cortex, cingulate gyri, ventromedial prefrontal cortex, and hippocampus during the same auditory recognition task. The present findings also resonate with additional prior research. The network identified by the first principal component, involving the auditory cortices and cingulate gyrus, aligns with ROI‐based studies on functional and structural connectivity, which have demonstrated strong links between these regions in both humans and animals.^[^ ^63^, ^64^, ^65^ ^]^ This supports their joint role in early auditory processing and sustained attention.^[^ ^66^, ^67^, ^68^ ^]^ Similarly, the network identified by the second principal component engaged regions typically associated with memory and prediction.^[^ ^69^, ^70^, ^71^ ^]^ Previous studies have shown that these memory‐related regions co‐activate in both structural and functional connectivity, reinforcing their role in comparing incoming sounds with stored memory traces.^[^ ^72^ ^]^ Crucially, unlike previous studies constrained to predefined ROIs, our findings reveal two whole‐brain networks directly engaged in the task. Here, the auditory cortex appears to serve a dual function: engaging with medial cingulate structures for fundamental auditory processing while also interacting with memory‐related regions for predictive computations.

To further clarify the spatial organization of these two principal networks, we conducted a gradient and clustering analysis of their voxel‐wise contributions. Inspired by similar techniques used in recent fMRI studies,^[^ ^51^, ^52^, ^53^ ^]^ we embedded the thresholded spatial maps of the first two MEG BROAD‐NESS components into a 2D space and applied unsupervised clustering. This procedure revealed a consistent and interpretable subdivision of voxel contributions across the brain. The optimal solution identified eight spatial clusters, each defined by a distinct pattern of co‐involvement in the two networks. This analysis confirmed the presence of voxels strongly engaged in both networks, particularly within the bilateral primary and secondary auditory cortices, reinforcing our interpretation of their shared role in both systems. We also observed voxels selectively contributing to only one network: the medial and posterior cingulate gyrus for Network #1, and the anterior cingulate and right hippocampus for Network #2. Interestingly, some voxels exhibited opposing contributions across the two networks. For example, voxels in the medial cingulate gyrus contributed positively to Network #1 but negatively to Network #2, while the reverse was observed in the anterior cingulate. This functional opposition underscores a clear division within the cingulate cortex, suggesting distinct subregions engaged in contrasting aspects of network dynamics. Additionally, the analysis revealed spatially coherent clusters of voxels with minimal or no contribution to either network, likely representing non‐task‐specific background activity. These regions encompassed much of the occipital lobe, unimodal sensory areas (e.g., visual and sensorimotor cortices), and frontal regions not implicated in the current task. These results offer a fine‐grained anatomical mapping of how large‐scale network participation is spatially distributed in the brain and support the interpretation that the two BROAD‐NESS networks reflect partially overlapping yet functionally dissociable systems operating simultaneously. Importantly, by leveraging the BROAD‐NESS analytical framework, we reveal network‐level organizational principles of auditory memory recognition that were not accessible with ROI‐based methods.

Second, our results relate to and extend the well‐known dual‐stream hypothesis, originally proposed for vision and later applied to auditory processing.^[^ ^73^, ^74^, ^75^, ^76^ ^]^ This framework posits two principal pathways: a ventral stream, leading from sensory areas to the medial temporal lobe and implicated in object recognition, and a dorsal stream, extending to the parietal lobe and primarily associated with spatial processing. Consistent with this hypothesis, one of the two networks identified in our study includes core regions of the ventral stream, such as the hippocampus, ventromedial prefrontal cortex, and inferior temporal cortex, supporting its role in recognition processes. Crucially, we demonstrate that this network not only aligns with the ventral stream but also exhibits clear evidence of predictive coding, encompassing both confirmed predictions and prediction errors unfolding over time. This suggests that predictive coding mechanisms are intrinsically linked with the role of the ventral stream in memory‐based auditory processing. Interestingly, our findings also indicate a second, distinct network that differs from the classical dorsal stream. Instead of engaging the parietal lobe, this alternative network is centered around the auditory cortices and medial cingulate gyrus. While this network also exhibits predictive coding features, showing differences between experimental conditions and signal peaks in response to deviations in melodies, the predictive coding effects are notably weaker than in the ventral‐stream‐aligned network. Instead, this network appears to be more closely related to the general processing of auditory stimuli, including an early N100 ERF component followed by a slower negative peak, which shows only limited variation across conditions. These results suggest a novel perspective: rather than a direct dorsal counterpart, we identify a ‘medial’ or ‘central’ stream that supports sustained auditory processing and possibly attention, while playing a comparatively reduced role in the computation of confirmed prediction and prediction error.

Third, a major contribution of this study is the demonstration that predictive coding during auditory memory recognition is embedded within large‐scale, orthogonal yet dynamically coordinated networks, rather than being confined to local circuits or pairwise interactions, as typically observed in previous research using small networks formed by, for example, two to six ROIs. While earlier studies have primarily described predictive coding in terms of error propagation within restricted pathways,^[^ ^20^, ^21^ ^]^ our findings suggest that this process is integrated into broader network‐level computations that simultaneously encode both confirmed predictions and deviations from expectation. This perspective shifts the focus from the conventional view of predictive coding as an exchange between discrete hierarchical levels toward a model in which distributed regions contribute in parallel to different aspects of prediction. The concurrent engagement of these networks implies that prediction and prediction error are not processed in isolation but unfold as part of a coordinated large‐scale neural mechanism. Moreover, the differential involvement of these networks across time suggests that predictive coding operates across multiple temporal scales, integrating rapid sensory updates with slower memory‐based processes. To further investigate this temporal organization, we embedded the time series of the two core BROAD‐NESS networks into a 2D phase space and applied RQA. This analysis revealed structured differences in temporal dynamics across experimental conditions. Notably, comparisons between the M condition and the deviant condition where variation occurred at the final tone (NT4) showed significantly higher recurrence metrics for M. These included mean diagonal line length, determinism, entropy, and trapping time, all indicative of more stable, predictable, and coherent neural trajectories during successful memory recognition. One plausible explanation for this selective effect is that, in NT4, the fifth tone disrupted a well‐formed perceptual and metrical structure, making the deviation especially salient and cognitively disruptive compared to other N sequence categories (i.e., NT1, NT2, NT3). We further examined the behavioral significance of these recurrence dynamics by correlating RQA metrics with task performance. Strikingly, six out of eight metrics showed consistent and significant associations with accuracy and RTs. The strongest effects were observed for determinism and mean diagonal line length, which index the extent and duration of repeated temporal patterns in the phase space, where higher values reflected more structured and recurrent brain activity, which in turn predicted better performance. Entropy, measuring the richness and diversity of these patterns, was also positively associated with performance, suggesting that more complex but still orderly dynamics support efficient prediction and memory recognition. Trapping time and laminarity, which quantify the duration of stable, unchanging states within the networks, were similarly beneficial. Conversely, divergence, reflecting sensitivity to initial conditions and neural instability, was negatively associated with both accuracy and speed, pointing to the detrimental role of disordered or chaotic network‐embedded dynamics. Together, these results highlight that temporally consistent, predictable, and information‐rich network trajectories are tightly linked to behavioral success in auditory memory recognition and predictive coding. By integrating this dynamical systems perspective with the BROAD‐NESS framework, we extend our central claim: predictive coding arises not only from the co‐activation of orthogonal large‐scale networks, but from their embedding in structured temporal trajectories that scaffold cognitive function. Phase space and RQA analyses enrich BROAD‐NESS by linking spatial decomposition with temporal recurrence, offering a powerful lens on how stable, coherent dynamics support memory‐based prediction. This approach refines models of predictive processing,^[^ ^77^, ^78^ ^]^ by grounding cognitive success in the structured dynamics of whole‐brain networks.

These three main insights were further enriched by analyses exploring the relationship between the brain networks' time series and individual differences in behavioral performance. Specifically, we examined how the temporal dynamics of the two networks identified through the BROAD‐NESS analytical pipeline related to three behavioral measures: (i) accuracy in the memory task during MEG recording, (ii) reaction times (RTs), and (iii) musical expertise. Notably, neural activity following the presentation of the memorized musical sequence showed significant correlations with accuracy in both brain networks. This suggests that this time window may reflect cognitive processes critical for decision‐making when participants were recognizing previously memorized sequences, rather than processing varied ones. Although correlations with RTs were less robust, they spanned a wider range of conditions, revealing weaker yet significant associations between network activity and response speed. This pattern indicates a more distributed involvement of these networks in modulating task performance based on temporal demands. Furthermore, musical expertise was significantly associated with prediction error responses in brain network #2, particularly when a deviant sound was introduced at positions three or four in the sequence. This finding highlights a connection between musical expertise and the neural processing of unexpected auditory events. This is consistent with previous literature showing enhanced neural responses in musicians and systematic differences between musicians and non‐musicians across both music‐specific and broader cognitive domains.^[^ ^6^, ^7^, ^79^, ^80^, ^81^, ^82^, ^83^, ^84^, ^85^, ^86^ ^]^

While our study focused on the two primary networks that explained the majority of the variance, the BROAD‐NESS analytical pipeline also revealed additional networks that were above chance level. Specifically, four smaller networks emerged, each accounting for a minor percentage of variance (4.16%, 1.30%, 0.99%, and 0.92%, respectively). These networks contained subsets of regions from the two primary networks but exhibited less interpretable activation patterns and time series. The less distinct temporal dynamics and activation patterns of these additional networks suggest that they likely reflect a minor proportion of residual activity mixed with noise, rather than major contributions to the predictive and memory processes.

Importantly, our findings on predictive coding for auditory sequences were enabled by the novel BROAD‐NESS analytical pipeline, which represents a significant advancement over traditional methods in cognitive neuroscience for studying brain networks. Therefore, it is essential to discuss them also in relation to specific methodological aspects and nuances (for further technical details on BROAD‐NESS, please refer to the Experimental Section and Supplementary Information). An essential remark is that traditional approaches to studying brain networks have typically relied on predefined ROIs, falling into three main categories: (i) pairwise connectivity without directionality (e.g. static functional connectivity,^[^ ^87^ ^]^ instantaneous phase synchronization^[^ ^88^ ^]^); (ii) pairwise connectivity with directionality (e.g. Granger causality,^[^ ^89^ ^]^ transfer entropy,^[^ ^90^ ^]^ partial directed coherence^[^ ^91^ ^]^); and (iii) functional network analyses constrained to predefined ROIs or parcellations (e.g. DCM,^[^ ^19^ ^]^ graph theory on connectivity matrices,^[^ ^92^ ^]^ hidden Markov models^[^ ^93^ ^]^). While these methods have yielded valuable insights, they are limited by their reliance on predefined brain regions, potentially overlooking key network dynamics. In particular, a‐priori parcellation imposes rigid spatial boundaries that may not align with the true functional organization of the brain, especially when networks overlap or dynamically reconfigure over time. This would be a crucial limitation in the context of predictive coding and memory processes, which likely involve flexible engagement of distributed regions that do not map cleanly onto anatomical or standard functional atlases. By forcing the data into predefined parcels, traditional approaches may obscure subtle yet meaningful interactions across voxel‐level signals, reducing sensitivity to emergent network‐level dynamics. In contrast, BROAD‐NESS operates on the entire source‐reconstructed MEG space, analyzing a high‐resolution voxel grid (8 mm) that preserves spatial information while leveraging the high temporal resolution inherent to MEG. By avoiding ROI‐based constraints,^[^ ^94^, ^95^, ^96^ ^]^ this approach fully exploits the advantages of MEG source reconstruction, allowing for a data‐driven exploration of the whole‐brain networks. Furthermore, BROAD‐NESS features a streamlined analytical pipeline that is both computationally efficient and based on only a few well‐supported assumptions: (i) brain voxels are modelled as a linear combination of sources mixed with noise, with networks emerging when voxels covary over time; (ii) principal components derived from BROAD‐NESS correspond to brain networks with interpretable dynamics; and (iii) variance explained by each component reflects the network's relative significance. Moreover, BROAD‐NESS is optimised for event‐related designs, effectively isolating transient brain networks involved in stimulus processing.

An additional advantage of the BROAD‐NESS framework is that, by leveraging PCA, it yields orthogonal components, a property that enhances the reliability and interpretability of the derived brain networks. Orthogonality ensures that each network captures distinct, non‐overlapping variance in the data, and that the associated time series are uncorrelated. This avoids redundancy and multicollinearity, improving the robustness of statistical comparisons across conditions or behavioral variables. Moreover, this is also conceptually compatible with theories of neural subspaces, where cognitive processes are represented within independent dimensions or trajectories of population‐level brain activity.^[^ ^97^ ^]^ Crucially, the orthogonal spatial filters obtained from group‐averaged data can be directly applied to individual participants and conditions without re‐estimating the decomposition, preserving consistency and reducing overfitting. Compared to non‐orthogonal methods such as ICA, which often lack component ordering and stability across runs, PCA thus offers a principled and computationally efficient way to extract meaningful large‐scale networks from complex, high‐dimensional neurophysiological data. To provide proper quantitative results supporting such reasoning, we also computed an ICA for detailed comparison with the BROAD‐NESS PCA approach. Overall, ICA returned results that were broadly consistent with those obtained through BROAD‐NESS, confirming the presence of network‐level modulations associated with auditory memory and predictive coding. However, the ICA‐derived components were generally more scattered and less interpretable, both in terms of their spatial activation patterns and their temporal dynamics. They also exhibited weaker and less consistent differences between experimental conditions. Furthermore, ICA's lack of an objective ranking metric, such as explained variance, complicates the identification of physiologically meaningful components, particularly at higher component counts where over‐separation can introduce algorithmic noise. In contrast, PCA‐based BROAD‐NESS leverages variance‐ordering to yield compact, stable, and interpretable network representations, while also facilitating principled estimates of network dimensionality. Our results also revealed a consistent linear decay in the correlation structure between ICA and PCA components. Across all ICA decompositions (ranging from two to 26 components), the first few ICA components consistently showed the highest similarity to the PCA‐derived networks, followed by a steady decline in correspondence. This evidence further highlights ICA's tendency toward less interpretable outputs, especially as component number increases. Nonetheless, ICA may still offer complementary value. For instance, in studies targeting finer‐grained subnetwork structure or individual variability, ICA could uncover functionally meaningful patterns less evident from PCA alone. For example, in our dataset, ICA suggested a tentative dissociation between four overlapping networks involved in auditory predictive processing and memory. Two networks, centered on the medial and anterior cingulate gyrus (ICA #3 and #6), were selectively engaged during conscious prediction error responses and exhibited slower temporal dynamics. A third, more left‐lateralized network involving the hippocampus and auditory cortex was associated with faster, sharper prediction error responses (ICA #8). A fourth network, partially overlapping with the third but extending into right‐hemispheric regions, showed dynamics more strongly linked to confirmed predictions rather than prediction errors (ICA #4). While these components were more fragmented, less stable and capable of differentiating experimental conditions than PCA ones, they may still reflect meaningful subcomponents of a broader predictive coding architecture. To conclude, while the PCA‐based BROAD‐NESS approach yields robust and highly interpretable results and remains the recommended method, incorporating a combined PCA–ICA workflow within the BROAD‐NESS pipeline could provide additional value. This hybrid strategy preserves the principled, variance‐based decomposition of PCA while allowing for optional, hypothesis‐driven or exploratory ICA analyses to probe potential subnetworks. Along these lines, although in this study we demonstrated the value of using PCA and additional analytical methods on source‐reconstructed MEG data, it is important to note that alternative decomposition methods may also prove valuable depending on the nature of the data and the research question. For example, Yi and colleagues^[^ ^98^ ^]^ developed a Bayesian non‐negative matrix factorization (NMF) framework for EEG, which enforces physiologically meaningful non‐negative activations and permits overlapping subnetworks, yielding complementary insights into brain network organization. Future work should build on these advances to explore how different decomposition approaches can be integrated or tailored to specific neuroscientific questions.

Beyond its immediate outputs, the PCA implementation in BROAD‐NESS offers a versatile foundation for further analyses within the same pipeline. For example, the brain networks derived through our approach can be combined with methods such as transfer entropy or DCM to investigate non‐linear interactions and causal relationships at the network level. Similarly, directionality analyses can be performed on the identified network time series, offering further insights into causal interactions. Additionally, although BROAD‐NESS does not assume recurrent states, multivariate RQA can be applied to its time series to infer potential trajectories within a state space defined by the orthogonal brain networks identified by the framework,^[^ ^99^, ^100^ ^]^ as demonstrated in the present study.

The time series output of BROAD‐NESS is also ideally suited for integration with behavioral and stimulus‐level variables, a strength already demonstrated in the present study. We successfully linked network activity to participants’ musical expertise, recognition accuracy, and reaction times, highlighting how BROAD‐NESS can reveal meaningful relationships between large‐scale neural dynamics and individual cognitive profiles. Because the method generates interpretable, condition‐specific time series across participants, it enables robust group‐level comparisons and behavioral correlations. For example, differences in network engagement could be related to measures such as sensitivity to sequence violations, general and system‐specific memory abilities, or individual differences along pitch perfection such as absolute or relative pitch skills. In future research, BROAD‐NESS could also support the integration of parametric stimulus features, such as tonal predictability, melodic complexity, or memory load, allowing researchers to examine how specific structural attributes modulate network responses. These future directions highlight how BROAD‐NESS not only provides novel insights in the current state, but also can be integrated with existing methodologies, paving the way for an even enriched understanding of the network dynamics underlying predictive coding.

In conclusion, our study provides novel insights into the large‐scale network organization of predictive coding in auditory memory recognition, revealing two concurrent whole‐brain networks with distinct functional responses and roles. By leveraging the BROAD‐NESS framework, we overcome previous methodological constraints and offer a fine‐grained, interpretable characterisation of predictive processing at the whole‐brain network level. Through the integration of phase space embedding and RQA, we further demonstrate how temporal dynamics reflect behavioral performance, while spatial gradients embedding analysis uncovers the fine structure of voxel‐level network contributions. Complementary ICA comparisons underscore the robustness and high interpretability of the PCA‐derived components. Taken together, this work provides both conceptual and methodological advancements. Conceptually, it refines current theories of memory and predictive coding by demonstrating that these processes are implemented through distributed, temporally embedded, and dynamically interacting brain networks. Methodologically, it introduces BROAD‐NESS, an integrated pipeline for deriving and analyzing the spatial and temporal organization of whole‐brain network activity, offering new tools for studying behaviorally relevant neural dynamics at high spatiotemporal resolution.

## 4 Experimental Section

### Participants

The participant sample comprised 83 volunteers [33 males and 50 females (biological sex, self‐reported)] aged between 19 and 63 years (mean age: 28.76 ± 8.06 years). Participants were recruited in Denmark, primarily from Western countries. Information on gender was not collected, as it fell outside the scope of this research. All participants were healthy, reported normal hearing, and had relatively homogeneous educational backgrounds. Specifically, 73 participants either held a university degree (bachelor's or master's, n = 54) or were currently enrolled as university students (n = 19). Of the remaining 10 participants, five held a professional degree earned after high school, and the other five had only completed high school. The study was approved by the Institutional Review Board (IRB) of Aarhus University (case number: DNC‐IRB‐2020‐006) and conducted in accordance with the Declaration of Helsinki – Ethical Principles for Medical Research. All participants provided informed consent prior to the experiment and received compensation for their participation.

### Experimental Stimuli and Design

In this study, we employed an old/new auditory recognition task during magnetoencephalography (MEG) recordings (Figure 1), as described in previous studies.^[^ ^7^, ^122^, ^123^, ^124^, ^125^, ^126^ ^]^ Participants first listened to a short musical piece twice and were asked to memorize it. The piece consisted of the first four bars of the right‐hand part of Johann Sebastian Bach's Prelude No. 2 in C Minor, BWV 847, where each bar contained 16 tones, totaling 64 tones. Each tone lasted ≈350 ms, making the total duration 22 400 ms. A final tone, lasting 1000 ms, was added for a sense of closure, bringing the full duration to 23 400 ms (23.4 s). The musical notation for the prelude is shown in Figure S1 (Supporting Information).

After memorizing the piece, participants were presented with 135 five‐tone musical excerpts, each lasting 1750 ms. They were asked to determine whether each excerpt belonged to the original music (‘memorized’ sequence [M], old) or was a variation (‘novel’ sequence [N], new). Among the excerpts, 27 were taken directly from the original musical piece, while the remaining 108 were variations. Figure S2 (Supporting Information) provides a visual representation of all the sequences used in the study.

The memorized sequences (M) were composed of the first five tones from the first three measures of the original piece, and each sequence was repeated nine times, totaling 27 trials. Novel sequences (N) were created by systematically varying the M sequences. For each of the three M sequences, nine variations were created by altering tones after the first (NT1), second (NT2), third (NT3), or fourth (NT4) tone, resulting in 27 variations per category and 108 novel sequences overall.

The variations followed several strategies. In some cases, the melodic contour was inverted, reversing the melodic direction of the original sequence. In other instances, the remaining tones were scrambled, or the same tone was repeated multiple times, sometimes varying only the octave. Another approach scrambled the intervals between tones. In most cases, the harmonic structure of the novel sequences was preserved relative to the original sequence. Further information about the sequences is reported in Bonetti et al.^[^ ^33^ ^]^

This procedure allowed us to explore the brain networks dynamics involved in recognizing previously memorized auditory sequences and in the conscious detection of sequence variations. The musical piece and sequences described above were generated as MIDI versions using Finale (version 26, MakeMusic, Boulder, CO) and presented through Psychopy v3.0.

### Old/New Paradigm Behavioral Performance

Behavioral data was collected during the recognition task in the MEG (old/new paradigm), including the number of correctly recognized trials and the corresponding reaction times. As the data was not normally distributed, we employed two independent Kruskal–Wallis H tests (a non‐parametric alternative to one‐way ANOVA) to assess whether performance differed across the five temporal sequence conditions (M, NT1, NT2, NT3, NT4). Post hoc pairwise comparisons were corrected for multiple testing using the Tukey–Kramer method.

### Neural Data Acquisition

The MEG recordings were conducted in a magnetically shielded room at Aarhus University Hospital (AUH), Denmark, using an Elekta Neuromag TRIUX MEG scanner with 306 channels (Elekta Neuromag, Helsinki, Finland). Data was collected at a sampling rate of 1000 Hz with an analogue filtering of 0.1 – 330 Hz. Prior to the recordings, participants’ head shapes and the positions of four Head Position Indicator (HPI) coils were registered relative to three anatomical landmarks using a 3D digitizer (Polhemus Fastrak, Colchester, VT, USA). This information was later used to co‐register the MEG data with anatomical MRI scans. Throughout the MEG recording, the HPI coils continuously tracked the position of the head, allowing for movement correction during analysis. Additionally, two sets of bipolar electrodes were used to monitor cardiac rhythm and eye movements, facilitating the removal of electrocardiography (ECG) and electrooculography (EOG) artefacts in the analysis phase.

MRI scans were obtained on a CE‐approved 3T Siemens MRI scanner at AUH. The data included structural T1 (mprage with fat saturation) scans, with a spatial resolution of 1.0×1.0×1.0 mm and the following sequence parameters: echo time (TE) of 2.61 ms, repetition time (TR) of 2300 ms, a reconstructed matrix size of 256 × 256, an echo spacing of 7.6 ms, and a bandwidth of 290 Hz/Px.

The MEG and MRI recordings were conducted on separate days.

### MEG Data Pre‐Processing

The raw MEG sensor data (comprising 204 planar gradiometers and 102 magnetometers) was initially pre‐processed using MaxFilter^[^ ^101^ ^]^ (version 2.2.15) to reduce external interferences. Signal space separation (SSS) was applied with the following MaxFilter parameters: downsampling from 1000 to 250 Hz, movement compensation via continuous HPI coils (default step size: 10 ms), and a correlation limit of 0.98 to reject overlapping signals between inner and outer subspaces during spatiotemporal SSS.

The MEG data was converted into Statistical Parametric Mapping (SPM) format and further processed and analysed using MATLAB (MathWorks, Natick, MA, USA), incorporating custom‐built scripts (LBPD, https://github.com/leonardob92/LBPD‐1.0.git) and the Oxford Centre for Human Brain Activity (OHBA) Software Library (OSL)^[^ ^102^ ^]^ (https://ohba‐analysis.github.io/osl‐docs/), which integrates Fieldtrip,^[^ ^103^ ^]^ FSL,^[^ ^104^ ^]^ and SPM^[^ ^105^ ^]^ toolboxes.

Next, the continuous MEG data underwent visual inspection via the OSLview tool to remove any large artifacts, though less than 0.1% of the collected data was discarded. Independent Component Analysis (ICA) was then used (with OSL implementation) to eliminate eyeblink and heartbeat artifacts.^[^ ^106^ ^]^ The ICA process involved decomposes the original signal into independent components and correlating them with the activity recorded by the electrooculography (EOG) and electrocardiography (ECG) channels.^[^ ^107^ ^]^ Components that showed a correlation at least three times higher than others were flagged as reflecting EOG or ECG activity. These flagged components were further validated through visual inspection, ensuring their topographic distribution matched typical eyeblink or heartbeat activity, and were subsequently discarded. The signal was then reconstructed using the remaining components.

Finally, the data was segmented into 135 trials (27 M, 27 NT1, 27 NT2, 27 NT3, and 27 NT4) and baseline‐corrected by subtracting the mean baseline signal from the post‐stimulus data. Each trial lasted 4500 ms (4400 ms of data and 100 ms of baseline).

### Source Reconstruction

MEG is a powerful tool for detecting whole‐brain activity with excellent temporal resolution. However, to fully understand the brain activity involved in complex cognitive tasks, it is essential to identify the spatial sources of this activity. This requires solving the inverse problem, as the MEG recordings reflect neural signals from outside the head but do not directly indicate which brain regions generated them. To address this, beamforming algorithms,^[^ ^101^, ^103^, ^104^ ^]^ using a combination of in‐house‐developed codes alongside the OSL, SPM, and FieldTrip toolboxes were employed. The procedure consisted of two main steps: (i) designing a forward model and (ii) computing the inverse solution.

First, a single‐shell forward model was constructed using an 8‐mm grid. This head model treats each brain source as an active dipole and describes how the activity of such a dipole would be detected across MEG sensors.^[^ ^108^, ^109^ ^]^ The MEG data was co‐registered with individual MRI T1‐weighted images, using the 3D digitizer information to align the data. When individual anatomical scans were unavailable, an MNI152‐T1 template with 8‐mm spatial resolution was used to compute the forward model.

Second, a beamforming algorithm was applied as the inverse model. Beamforming uses a set of weights applied to different source locations (dipoles) to isolate the contribution of each brain source to the activity recorded by the MEG sensors.^[^ ^110^, ^111^, ^112^ ^]^ This was done for every time point of the recorded brain data, allowing us to reconstruct the spatial sources of the MEG signal. The detailed steps for implementing the beamforming algorithm are provided below.

The data recorded by the MEG sensors (*B*) at time *t*, can be described by the following Equation (1):

(1) $$B_{\left(t\right)}=L*Q_{\left(t\right)}+\varepsilon$$

where *L* is the leadfield model, *Q* is the dipole matrix carrying the activity of each active dipole (*q*) at time *t*, and *Ɛ* is noise (for details, see Huang et al.^[^ ^109^ ^]^) To solve the inverse problem, *Q* must be estimated for each *q*. In the beamforming algorithm, a series of weights are computed to describe the transition from MEG sensors to the active dipole *q*, independently for each time point. This is reported in Equation (2):

(2) $$q_{\left(t\right)}=W^{T}*B_{\left(t\right)}$$

Here, the superscript *T* refers to transpose matrix. To compute the weights (*W*), matrix multiplication between *L* and the covariance matrix of MEG sensors (*C*) is performed. Importantly, the covariance matrix *C* was computed on the signal after concatenating the single trials of all experimental conditions. For each dipole *q*, the weights (*W_q_*) were computed as shown in Equation (3):

(3)

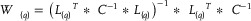




The computation of the leadfield model *L* was performed for the three main orientations of each dipole.^[^ ^110^ ^]^ Before computing the weights, to simplify the beamforming output,^[^ ^113^, ^114^ ^]^ the orientations were reduced to one using the singular value decomposition algorithm on the matrix multiplication reported in Equation (4):

(4) $$L=svd{\left(l^{T}*\;C^{-1}*l\right)}^{-1}$$

Here, *l* represents the leadfield model with the three orientations, while *L* is the resolved one‐orientation model that was utilized in Equation (3).

Once computed, the weights were normalized for counterbalancing the reconstruction bias toward the center of the head^[^ ^103^, ^115^, ^116^, ^117^ ^]^ and applied to the neural activity averaged over trials, independently for each time point (Equation 2) and experimental condition. This procedure returned a time series for each of the 3,559 brain sources.^[^ ^103^, ^108^ ^]^

### Principal Component Analysis (PCA), Monte‐Carlo simulations (MCS) and Randomization

PCA was applied to the 3,559 reconstructed brain sources time series to disentangle broadband brain networks operating simultaneously. PCA is a dimensionality reduction method that transforms data into new variables, or principal components, which account for the most variance within the dataset. This is achieved by computing the eigenvectors and eigenvalues of the data's covariance matrix and then projecting the data onto the directions of maximum variance. Traditionally, PCA simplifies high‐dimensional data while retaining the most important information.

In this study, however, PCA was applied to a dense set of MEG‐reconstructed brain data at the voxel level (3559 brain voxel time series). The goal was not just to reduce the dimensionality but to identify brain networks (corresponding to the PCs) through the application of PCA on the 3559 brain voxel time series.

In short, PCA operates as follows. First, the data was centered by subtracting the mean of each brain voxel timeseries from the dataset *X*, as represented by Equation (5):

(5) $$\bar{X}=X-\mu$$

where μ is the mean vector of *X*.

Then, the covariance matrix *C* is computed on the centered data, as shown in Equation (6):

(6) $$C=\frac{1}{n-1}\;{\bar{X}}^{T}\bar{X}$$

where *n* is the number of data points.

Then the eigenvalue equation for the covariance matrix is solved to find eigenvalues and eigenvectors, Equation (7):

(7) CW=WΛ

where *W* is the eigenvector matrix (set of weights for each principal component) and Λ is the diagonal matrix of the corresponding eigenvalues (which indicate the amount of variance explained by each component).

Then, the eigenvectors *w* were used to compute the activation time series of each brain network *y* by multiplying them by the original data *X*, as shown in Equation (8):

(8) $$y\;=w^{T}X$$

Furthermore, the spatial projection of each component (spatial activation patterns *a*) was computed in brain voxel space. This was done by multiplying the weights of the analysis (the eigenvectors *w* in this case) by the covariance matrix *C*, as shown in Equation (9):

(9) $$a=w^{T}C$$

Interestingly, while previous studies on interpreting weights from multivariate analyses in MEG data have recommended calculating spatial activation patterns,^[^ ^118^ ^]^ in this case, the relative contribution of each brain voxel to the network remains the same, whether using the direct eigenvector *w*​ or computing the spatial activation patterns.

It is important to note that PCA can be computed using different mathematical approaches that yield equivalent results. A commonly used alternative is to apply Singular Value Decomposition (SVD)^[^ ^119^ ^]^ directly to the mean‐centered data matrix. This approach was often preferred in practice because it was more numerically stable and computationally efficient, especially for high‐dimensional data (for instance, this was the solution currently implemented in the ‘pca’ function in MATLAB). However, since the SVD‐based solution ultimately produces the same principal components and scores as the covariance‐based eigen decomposition described above, it was chosen to retain the latter formulation. This choice was motivated by clarity and pedagogical value, as it offers a more intuitive explanation of the underlying principles and aligns directly with the Generalized Eigen decomposition (GED) procedure employed in the FREQ‐NESS analytical pipeline.^[^ ^50^ ^]^ Highlighting this parallel helps readers better appreciate both the similarities and critical distinctions between BROAD‐NESS and FREQ‐NESS.

Importantly, as detailed in the following section, the PCA procedure was applied across various scenarios to ensure a comprehensive evaluation of the algorithm's performance. This included performing PCA independently for each participant and condition, as well as on data averaged across participants and conditions.

### Statistical analysis

After computing PCA and the time series of the brain networks for each participant and experimental condition, statistical analyses were conducted by contrasting the time series of the previously memorized versus the varied melodies. This approach was consistent with our earlier study that focused solely on brain ROIs.^[^ ^33^ ^]^ Specifically, a two‐sided *t*‐test for each time point across all combinations of M versus N melodies (i.e., M versus NT1, M versus NT2, M versus NT3, M versus NT4) was performed. Subsequently, False Discovery Rate (FDR) corrections were applied to account for multiple comparisons.

To strengthen the robustness of the findings and offer deeper insights into the process of contrasting time series of brain networks, it was implemented and compared three alternative methods for correcting for multiple comparisons. This analysis specifically focused on the contrast between M versus NT1.

First, the Bonferroni correction was applied, which was a stringent method that adjusts the significance level (𝛼 = 0.05) by dividing it by the number of tests performed, thereby reducing the likelihood of false positives.

Second, a cluster‐based permutation test was used, as described by Maris and Oostenveld.^[^ ^120^ ^]^ Initially, statistical tests were conducted on the original data, as described earlier. Then it identified clusters of neighboring significant time points by applying a threshold to the statistical results (α = 0.05). Next, 1000 permutations were performed, where, for each permutation, the labels of the two experimental conditions were shuffled for each participant, and the statistics were recalculated. For each permutation, the maximum cluster size of the significant time points was computed, mirroring the approach used for the original data. This yielded a reference distribution of significant clusters, which it compared with the clusters from the original data. Clusters in the original data were deemed significant only if their sizes exceeded the maximum cluster size observed in the permuted data at least 99.9% of the time.

Third, a 1D cluster‐based MCS (MCS, α = .05, MCS *p*‐value = 0.001) was conducted, as detailed in the previous studies.^[^ ^28^, ^29^, ^31^, ^32^, ^33^, ^121^, ^122^, ^123^ ^]^ This approach involved identifying clusters of significant neighboring time points obtained from the *t*‐tests described above and then performing 1000 permutations to randomise these significant values. For each permutation, we extracted the maximum cluster size to establish a reference distribution. The original clusters were considered significant if they were larger than 99.9% of the clusters obtained from the permuted statistical results. This method is similar to the cluster‐based permutation test but should be regarded as an empirical approach. Its primary advantage lies in its computational efficiency, delivering results much faster than the permutation test as it does not require recalculating statistics. Instead, it works directly with clusters formed from the statistics computed on the original data. However, a limitation is that this empirical method does not adhere to classical inferential statistical principles. In our analysis, we clarify how this approach relates to the permutation test, highlighting their respective advantages and limitations in the context of statistical testing for brain data time series. The statistical analyses were computed in MATLAB, using the standardized routines described above. All analyses involved the full sample of 83 participants.

### Phase Space and Recurrence Quantification Analysis (RQA) of PCA‐Derived Networks

To investigate the temporal dynamics and recurrence properties of the brain networks identified via PCA‐based BROAD‐NESS, a phase space and recurrence quantification analysis (RQA) was conducted ^[^ ^99^, ^124^ ^]^ on the time series of the two main PCA components (brain networks). This approach allows for the examination of the system's trajectory in a low‐dimensional temporal space, revealing underlying temporal structure that may not be evident in the original signal.

2D phase space trajectories were first constructed by treating the time series of the first two BROAD‐NESS networks as orthogonal axes. For each participant and experimental condition, the time‐resolved trajectory in this space was computed. Each point in the resulting scatterplot represents the state of the brain at a given time point, embedded in the space defined by the two dominant networks. Next, recurrence plots (RPs) were calculated for each participant and condition. RPs reflect the degree to which the system re‐enters similar network states over time.^[^ ^99^, ^124^ ^]^ Specifically, for each pair of time points, we computed the Euclidean distance between their respective phase space coordinates. Recurrence matrices were thresholded using a fixed ε‐radius criterion (10% of the maximum distance) to obtain binary plots, where a value of 1 indicates a recurrence.^[^ ^99^, ^124^ ^]^

From the thresholded RPs, eight standard metrics were extracted, commonly used in nonlinear time‐series and brain dynamics research:

1. Recurrence Rate (RR): proportion of recurrent points, indexing overall recurrency of the system.
2. Mean Diagonal Line Length (L): average length of diagonals, indicating temporal predictability.
3. Determinism (DET): proportion of recurrence points forming diagonal structures, reflecting the stability of the system trajectories.
4. Entropy (ENTR): Shannon entropy of diagonal lengths, indexing complexity of the system's evolution.
5. Trapping Time (TT): average vertical line length, reflecting temporal persistence in a given state.
6. Laminarity (LAM): fraction of recurrence points forming vertical lines, reflecting intermittency.

Maximal Laminar Duration (Vmax): length of the longest vertical line (i.e. period of state stability).

Divergence (DIV): inverse of the longest diagonal line, linked to system sensitivity and divergence in state space.

These metrics were computed independently for each participant and condition. Two‐sided *t*‐tests were performed comparing the memorized condition (M) with each novel tone condition (NT1, NT2, NT3, NT4), separately for each metric. *p*‐values were corrected for multiple comparisons using FDR, and only statistically significant results after correction were included in visualization and further interpretation.

### Correlation of Recurrence Metrics with Behavioral Performance

To examine the relationship between the dynamical properties of brain network trajectories and behavioral performance, we computed correlations between recurrence metrics and individual task outcomes. Specifically, we investigated whether the average values of the eight recurrence quantification metrics were predictive of two key behavioral measures from the memory task: overall accuracy and mean reaction time (RT). For accuracy, raw values were extracted from the five task conditions (M, NT1–NT4) and averaged across conditions to obtain a single accuracy score per participant. RTs were similarly averaged across the same five conditions. Recurrence metrics were also averaged across conditions for each participant, yielding a single value per metric per individual. For each metric, Spearman's rank‐order correlation was then computed with both accuracy and RT scores across participants. This non‐parametric approach was selected to account for potential deviations from normality in behavioral data distributions. To correct for multiple comparisons, FDR correction was applied to the sets of *p*‐values obtained from both accuracy and RT analyses (n = 16).

### Spatial Gradients Embedding and Clustering Analysis of Network Topographies

To examine the spatial organization of the dominant brain networks identified through BROAD‐NESS, we performed a spatial gradient embedding and clustering analysis on their voxel‐wise activation maps. This analysis was conducted on the data averaged across participants to obtain a robust, group‐level representation of network topographies. It was focused on the first two principal components (PC#1 and PC#2), which represented the most explanatory BROAD‐NESS‐derived brain networks. For each component, the voxel‐wise spatial weights by setting values between mean ± 1 standard deviation to zero were thresholded. This allowed to retain only the most strongly contributing voxels. These thresholded values were then used to define a 2D embedding space, where each voxel's position was determined by its weight on PC#1 (*x*‐axis) and PC#2 (*y*‐axis). This provided a gradient‐like spatial representation, capturing how individual voxels contributed to the two brain networks. To identify spatially coherent voxel clusters within this 2D space, we applied k‐means clustering^[^ ^125^ ^]^ to the embedded voxel coordinates. Prior to clustering, the data were z‐scored to normalize both axes and ensure equal weighting of the two components. For each value of *k* (ranging from 2 to 20),^[^ ^126^ ^]^ we computed the k‐means clustering with 100 repetitions with different random initializations to minimize the effect of convergence to local minima.^[^ ^125^ ^]^ To determine the optimal number of clusters, we computed the silhouette coefficient^[^ ^127^ ^]^ for each clustering solution. This metric assesses how well‐separated the resulting clusters are by comparing the average distance between points within the same cluster to the average distance between points in neighboring clusters. Higher silhouette values indicate better‐defined, more distinct clusters. However, due to the stochastic nature of k‐means initialization, the silhouette scores can vary slightly across runs. To account for this variability and ensure robust estimation of the optimal number of clusters, we computed silhouette scores 1,000 times. The final clustering solution was selected based on the number of clusters that was most frequently associated with the highest silhouette score across these repetitions. This method provided a robust and data‐driven way to quantify the spatial gradients underlying BROAD‐NESS‐derived network topographies. Crucially, it enabled the identification of consistent and interpretable voxel clusters, including those that predominantly contributed to one network, those shared between both, and those with negative or minimal contributions. This approach offers a principled means to uncover the fine‐grained spatial architecture of large‐scale functional brain networks.

### Behavioral Performance and Brain Network Activity

To further examine the relationship between behavioral performance, individual differences, and the brain networks identified in the present study, we conducted correlation analyses between the time series of the two networks extracted using our BROAD‐NESS analytical pipeline and three behavioral measures: (i) accuracy on the memory task during MEG recording, (ii) RTs, and (iii) musical expertise. Multiple comparisons were controlled using the false discovery rate (FDR) method.

### Independent Component Analysis (ICA) and Comparison with BROAD‐NESS

To further evaluate the robustness and functional interpretability of the BROAD‐NESS decomposition, an ICA was conducted using the JADE (Joint Approximate Diagonalisation of Eigenmatrices) algorithm.^[^ ^128^, ^129^ ^]^ ICA was applied to the source‐reconstructed MEG data averaged across all participants and experimental conditions, exactly as previously done for PCA. The number of components was fixed at 14, corresponding to the number of PCA components required to explain 95% of the total variance.

JADE is a widely used algorithm that separates statistically independent sources by exploiting fourth‐order cumulant information. Unlike many other ICA approaches, JADE is deterministic, meaning that it produces identical results across repeated runs on the same data. This property improves reproducibility and avoids issues of stochastic variation in component estimation, making it particularly suitable for validation analyses.

After computing the ICA spatial filters, network time series were reconstructed independently for each participant and each experimental condition by projecting the source‐reconstructed data onto the ICA filters, following the same procedure used in BROAD‐NESS. Spatial activation patterns for each independent component were computed by multiplying the ICA spatial filters by the covariance matrix of the MEG source‐reconstructed voxel data. This step mirrors the PCA approach and is recommended in multivariate brain analysis to obtain interpretable topographical maps.^[^ ^118^ ^]^

Statistical comparisons were then performed by contrasting the ICA time series across conditions (M vs each of the variation types: NT1, NT2, NT3, NT4) using two‐sided *t*‐tests and applying FDR correction, identical to the procedure used for the PCA‐derived BROAD‐NESS components.

To assess the degree of similarity between ICA‐ and PCA‐derived networks, Pearson correlation coefficients between the ICA time series and the time series of the two main brain networks (components) identified via PCA in BROAD‐NESS were computed. These correlations were calculated independently for each experimental condition, and the resulting correlation coefficients were averaged across conditions to facilitate summary and visualization. Finally, the ICA decomposition was repeated while systematically varying the number of extracted components from 2 to 26. For each decomposition, the correlation between the resulting ICA time series and the BROAD‐NESS PCA time series was computed, allowing to characterize how similarity trends evolved as a function of ICA dimensionality.

### Data Randomizations and Brain Networks

Finally, it was aimed to systematically assess how the sensitivity of BROAD‐NESS was influenced by the temporal and spatial dimensions of latent brain networks. To achieve this, the spatial and temporal organization of the data matrix (brain voxels x time points) was independently disrupted by implementing two types of randomizations. The randomization strategies were designed as follows: (i) space randomization, where the voxel indices were shuffled row‐wise; and (ii) time randomization, where the time indices were shuffled independently for each brain voxel. To evaluate the stability and robustness of the procedure, each randomization strategy was executed 100 times. The same statistical testing described for the original data was also performed for both randomization strategies.

Additionally, the second randomization was employed in a Monte Carlo simulation (MCS) solution. In this case, the variance explained by the first component of the PCA computed on the randomized data for each of the 100 permutations was retained. This produced a reference distribution of the variance explained by the first component in the randomized data. This reference distribution was then compared to the variance explained by the components in the original data, which were deemed significant only if they explained a greater variance than the highest explained variance in the reference distribution.

Time series and spatial activation patterns of the brain networks that were significant following the MCS approach are illustrated in Figure 2 and Figure S3 (Supporting Information), while the selective effects of each randomization strategy are presented in Figures S5–S7 (Supporting Information).

To further validate the effectiveness and distinct impact of the two randomization strategies, a set of complementary analyses designed to quantify the degree of disruption introduced by each approach was conducted. For each original and randomized dataset, static functional connectivity (FC) matrices using Pearson's correlation and applied Fisher's z‐transformation^[^ ^130^ ^]^ to normalize the correlation values was computed. The upper triangular portions (excluding the diagonal) of each z‐FC matrix were then vectorized to assess unique pairwise connectivity patterns. To evaluate the degree of disruption, similarity scores between each randomized FC and the original FC using Pearson's correlation was calculated. All pairwise similarities across datasets were also computed and projected the entire set of FC matrices into a low‐dimensional space using a PCA‐based approach inspired by Cov‐STATIS.^[^ ^131^ ^]^ To ensure computational efficiency, the number of permutations in Cov‐STATIS was limited to 50. These analyses revealed that both randomization strategies effectively altered the original FC structure, but with distinct profiles. Temporal randomization led to a complete loss of temporal structure, producing FC matrices that were highly uncorrelated with the original and among each other. In contrast, as expected, spatial randomization resulted in FC matrices that were still largely different from the original but still retained a modest degree of internal structure. Together, these additional evaluations support the validity of the randomization procedures and illustrate how the BROAD‐NESS pipeline relates to specific disruptions in spatiotemporal structure.

### Comparing Different PCA Computations

To enhance the robustness of our work and provide greater insight into the subtle differences associated with the computation of PCA on neural data, a crucial aspect relevant to experimental settings was examined. Specifically, when multiple experimental conditions are present, it is essential to determine whether it is more beneficial to compute PCA on individual conditions or on aggregated conditions (e.g., data averaged over conditions).

To address this, both approaches were computed and compared the results. Specifically, PCA was first conducted on the data averaged across conditions and then reconstructed the time series. Subsequently, PCA was computed independently for each condition. This allowed us to compare the brain networks time series of the conditions computed in the two approaches and assess any difference.

A similar consideration applies to the multiple participants in this study, where three possible approaches were explored for computing PCA. The first approach involved performing PCA on the average across participants, followed by reconstructing the time series independently for each participant using the weights obtained from this analysis. The second approach entailed performing PCA independently for each participant along with their respective time series. Finally, the third approach consisted of computing PCA on the concatenated data from all participants, and then deriving the time series independently for each participant. All three approaches were implemented and compared the results.^[^ ^33^ ^]^

## Conflict of Interest

The authors declare no conflict of interest.

## Author Contributions

L.B., G.F.R., M.H.A., and M.R. conceived the hypotheses; L.B. designed the study; L.B., M.L.K., C.T., and P.V. recruited the resources for performing data collection and analysis; L.B., G.F.R., and F.C. collected the data; L.B., G.F.R., M.H.A., C.M., and M.R. performed pre‐processing, statistical analysis, and linear decomposition of the neural signal; L.B., C.M., M.H.A., and M.R. developed the code for the BROAD‐NESS framework; M.L.K., C.T., P.V., and M.R. provided essential help to interpret and frame the results within the neuroscientific and analytical literature; M.H.A. and L.B. wrote the first draft of the manuscript, which was primarily integrated by M.R. and G.F.R The figures were prepared by L.B., G.F.R. and C.M. All the authors contributed to and approved the final version of the manuscript.

## Data and Code Availability

The BROADNESS Toolbox is available at the following link: https://github.com/leonardob92/BROADNESS_MEG_AuditoryRecognition/tree/main/BROADNESS_Toolbox. The complete pipeline for the study can be found here: https://github.com/leonardob92/BROADNESS_MEG_AuditoryRecognition/tree/main. The dataset used in this study (and suitable for testing the BROADNESS Toolbox) is available here: https://doi.org/10.5281/zenodo.17048137.

## Supporting information



Supporting Information

Supporting Information

Supporting Information

Supporting Information

Supporting Information

Supporting Information

Supporting Information

## Data Availability

The authors declare no competing interests.
