# Supplementary material for: BROAD‐NESS Uncovers Dual‐Stream Mechanisms Underlying Predictive Coding in Auditory Memory Networks
Source: Adv Sci (Weinh). 2025 Sep 29;12(44):e07878. doi: 10.1002/advs.202507878 (PMC12667469; doi:10.1002/advs.202507878)
Supplement: Supplementary file 1 — Supporting Information [file ADVS-12-e07878-s004.pdf]

Supplementary Information for

BROAD-NESS uncovers dual-stream mechanisms underlying predictive coding in auditory memory networks

Leonardo Bonetti [1,2,3], Gemma Fernández-Rubio [1], Mathias H. Andersen [1,4], Chiara Malvaso [5], Francesco Carlomagno [1,6], Claudia Testa [5], Peter Vuust [1], Morten L. Kringelbach [1,2,3], Mattia Rosso [1,7]**

[1] Center for Music in the Brain, Department of Clinical Medicine, Aarhus University & The Royal Academy of Music, Aarhus/Aalborg, Denmark

[2] Centre for Eudaimonia and Human Flourishing, Linacre College, University of Oxford, Oxford, United Kingdom

[3] Department of Psychiatry, University of Oxford, Oxford, United Kingdom

[4] Danish Research Centre for Magnetic Resonance, Hvidovre, Denmark

[5] Department of Physics and Astronomy, University of Bologna, Bologna, Italy

[6] Department of Education, Psychology, Communication, University of Bari Aldo Moro, Italy

[7] IPEM Institute for Systematic Musicology, Ghent University, Ghent, Belgium

*Corresponding authors: leonardo.bonetti@psych.ox.ac.uk (L.B.); mattia.rosso@clin.au.dk (M.R.)

Co-authors contacts:
gemmafr@clin.au.dk
mathiasha@drcmr.dk
chiara.malvaso@studio.unibo.it
calumaxs@gmail.com
claudia.testa@unibo.it
petervuust@gmail.com
morten.kringelbach@psych.ox.ac.uk

ORCID:
https://orcid.org/0000-0001-9983-3819

Additional discussion and clarifications of the BROAD-NESS analytical pipeline

In its essence, the estimation of the brain networks implemented in BROAD-NESS presents three key advantages over previous approaches.

First, it does not rely on predefined ROIs, as it operates on the entire source-reconstructed MEG space. This comprehensive approach, applied to the 3559-voxel grid with 8 mm resolution, is considered optimal for balancing spatial detail with the actual resolution of MEG data. By avoiding the reduction of data to predefined ROIs, as in traditional methods (e.g.[i-iii]), BROAD-NESS preserves spatial information which enables the embedding of spatial gradients, fully leveraging the benefits of MEG source reconstruction. Unlike ROI-based approaches that require a priori selection of brain regions, BROAD-NESS analyses all available data in voxel space, allowing for a fine-grained and data-driven exploration of brain activity.

Second, BROAD-NESS presents significant advantages in terms of simplicity and efficiency. It relies on a few well-supported assumptions, leading to results that are straightforward to interpret. The mathematical foundations of BROAD-NESS, which include covariance and eigenvector solutions, key elements of PCA, are relatively simple and less susceptible to artifacts compared to more complex signal processing techniques. Additionally, BROAD-NESS is extremely fast to compute, making it highly efficient for a variety of event-related paradigms, even with limited computational power or for large datasets. These unique features distinguish BROAD-NESS from other methods with similar aims and facilitate its integration with existing approaches. For instance, (i) BROAD-NESS can be effectively integrated with existing methods typically applied to brain ROIs to investigate non-linearity in the brain. While BROAD-NESS does not directly assess non-linear relationships, it disentangles simultaneous brain networks, which can then be examined for non-linearity using techniques such as RQA[iv,v], TE[vi], and DCM[vii]. These methods can be applied to the time series generated by BROAD-NESS, allowing for the exploration of non-linear interactions between entire brain networks rather than isolated ROIs. Furthermore, BROAD-NESS typically identifies a few large brain networks by focusing on a small number of principal components that explain most of the variance in the data. In this study, for instance, we identified two key networks accounting for 88% of the total variance. This streamlined approach not only reduces the computational demands for analyses like RQA[iv,v], TE, and DCM by retaining only the key data but also addresses the common challenge of defining ROIs, which often leads to excluding valuable data. Importantly, (ii) BROAD-NESS does not assume recurrent states in brain activity, in contrast to approaches like HMMs[viii,ix] and LEiDA[x]. However, while recurrent states are not the primary focus here, they can still be inferred through multivariate recurrence analysis of the time series generated by BROAD-NESS[xi,xii]. Similarly, (iii) while BROAD-NESS does not directly infer directionality or causality between brain networks, it provides time series data that can be further analysed using

existing methods designed for such purposes, like GC[xiii] or TE. In summary, while BROAD-NESS does not provide direct information regarding non-linearity, recurrent states, or directionality, it offers essential insights without assumptions about the spatial extent of the brain networks involved, paving the way for further examination of these metrics directly at a network level.

Third, BROAD-NESS effectively applies PCA to MEG source-reconstructed data in event-related designs to derive brain networks. Remarkably, BROAD-NESS retains both temporal resolution, similar to previous EEG studies computing dimensionality reduction at the scalp-level with PCA[xiv-xix] (for a review see[xx]), and, to a reasonable extent, spatial resolution, comparable to fMRI studies that applied ICA and PCA at the brain voxel level[xxi-xxv] to derive resting state brain networks over long recordings. The implementation of PCA in BROAD-NESS addresses limitations inherent to both methods, namely the limited interpretability of MEG sensors and the slower temporal dynamics of fMRI. By achieving an optimal trade-off between temporal and spatial resolution, our approach enables the identification of transient, rapid networks recruited for specific functions related to stimulus processing, while maintaining physiological interpretability.

Lastly, a few technical considerations warrant attention when applying PCA across multiple participants. In our study, we compared outputs from PCA applied to data averaged across all participants with PCA computed independently for each participant, followed by averaging the outputs, and with PCA applied to concatenated participant data. Although these approaches yielded coherent results, they differed significantly in clarity. PCA on averaged data produced cleaner and more interpretable results, exhibiting a sharper exponential decline in the variance explained by each principal component. In contrast, PCA on single participants and on concatenated data revealed potentially nuanced but less distinct patterns. Essentially, this discrepancy can be interpreted in two ways: (i) PCA applied to individual participants (either single participants or concatenated data) may capture more detailed information regarding inter-individual differences in brain networks, suggesting that the additional information retained is relevant to brain processes; or (ii) PCA on individual participants is simply decomposing a noisier matrix, resulting in less refined patterns and obscuring the clarity of the actual brain information. While we cannot conclusively determine which interpretation is correct, the tests conducted in this study, alongside previous recommendations for event-related designs in neurophysiology[xxvi] favour averaging across participants before computing PCA. This approach effectively reduces noise from both environmental factors and individual variability, thereby enhancing generalisability across the population. Notably, this approach contrasts with the strategy employed in FREQ-NESS[xxvii], where GED is computed independently for each participant using continuous data without averaging. This is essential to maintain the frequency-resolved content of the signal, which would otherwise be suppressed by averaging[xxvii]. In contrast, BROAD-NESS focuses on broadband networks and leverages time-locked averaging to maximise

the signal-to-noise ratio of event-related brain activation, resulting in considerably higher variance explained by the relevant networks.

To further explore the effects of averaging on our analysis, we applied PCA to either the data averaged across experimental conditions or to each condition separately. The results from these two approaches were nearly overlapping, indicating that the different conditions engaged almost identical brain networks. Importantly, computing PCA on the averaged data allowed us to properly derive the time series for the different conditions, enabling correct statistical testing. However, this approach is only valid if the experimental conditions activate similar brain networks; if they instead recruit significantly different networks, averaging is not appropriate. Thus, no definitive recommendation can be made, and the choice between these two alternative solutions should depend on the specific experimental protocol and hypotheses.

Finally, randomising the order of brain voxels, as observed in the evaluation of FREQ-NESS[xxvii], did not impact the variance explained or the resulting brain networks time series in this study; however, it did alter the spatial activation patterns. In contrast, randomising the data order over time disrupted the relationships within the data, resulting in significant reduction of the slope of the exponential decay of the variance explained by the principal components. It did also disrupt the clarity of the brain networks time series and the spatial activation patterns, further demonstrating the effectiveness of BROAD-NESS on real neural data.

In conclusion, beyond the main results about predictive coding described in our work, we also showed why and how BROAD-NESS can be successfully applied to a diverse range of event-related datasets. Expanding its use to explore various sensory domains and cognitive processes could indeed provide valuable insights into the brain networks underlying these events. Future research is also called to systematically compare BROAD-NESS with traditional ROI-based methods to determine whether the networks observed are merely a summation of ROI activations or if they reveal higher-order interactions, as suggested by the current findings. Such comparisons could elucidate whether the networks identified by BROAD-NESS reflect more complex neural dynamics that extend beyond the capabilities of the single brain ROIs. Additionally, as mentioned in the main text of the paper, BROAD-NESS is well-suited for integration with existing methods to examine directionality, recurrence, and non-linear interactions among brain networks. While some of these solutions are currently implemented in the BROAD-NESS toolbox, additional methods should be explored by future research. Finally, significant advancements within the NESS framework can also be made by investigating induced responses in event-related designs, enhancing our understanding of oscillatory brain behaviour and providing deeper insights into the temporal dynamics of cognition and its underlying neural mechanisms.

Supplementary figures

The same supplementary figures are available in high resolution at the following link:
https://doi.org/10.5281/zenodo.17048137

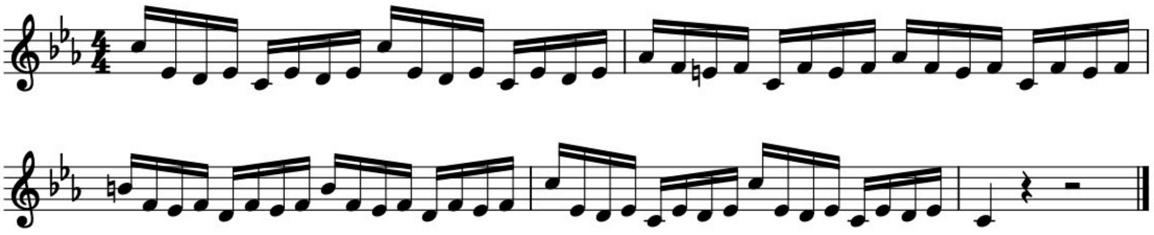

Figure S1. Musical piece used during the encoding phase of the auditory old/new paradigm
In the initial stage of the auditory old/new paradigm, participants listened to a brief musical piece twice and were instructed to memorise it as thoroughly as possible. The piece comprised the first four bars of the right-hand section of Johann Sebastian Bach's Prelude No. 2 in C Minor, BWV 847. Each bar contained 16 notes, resulting in a total of 16 x 4 = 64 notes. Each note lasted approximately 350 ms, amounting to a total duration of 22,400 ms. Additionally, to create a sense of musical closure, we included a final note after the four bars that lasted 1,000 ms. Consequently, the total duration was 23,400 ms, which equals 23.4 seconds. This figure presents the piece in musical notation.

Melody 1

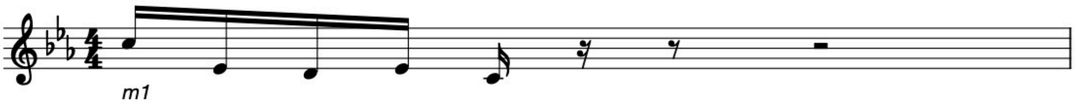

Inverted melodic contour I

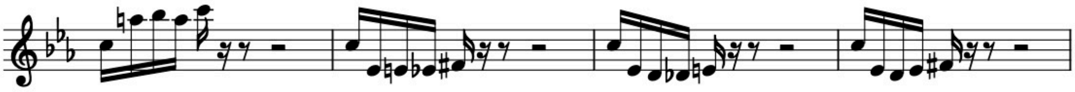

Same tone scrambled I

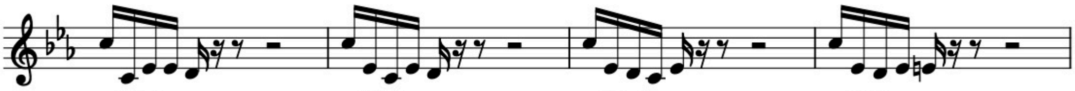

Same tone scrambled II

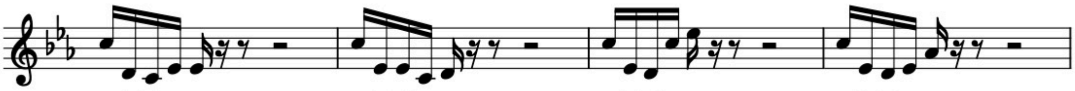

Same tone I

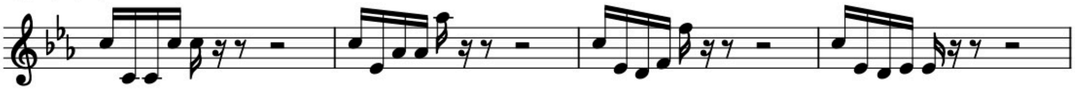

Same tone II

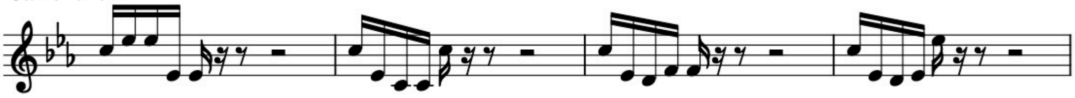

Scrambling intervals

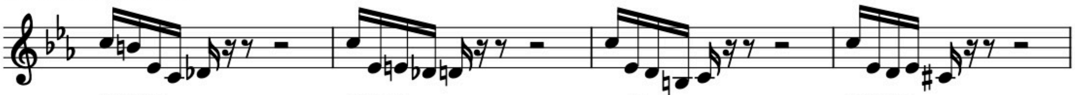

Inverted melodic contour II

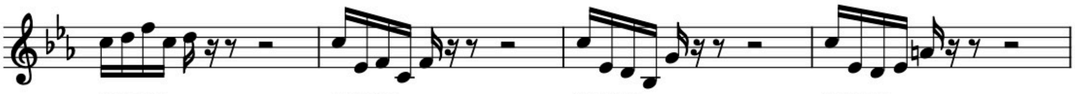

Same tone scrambled III

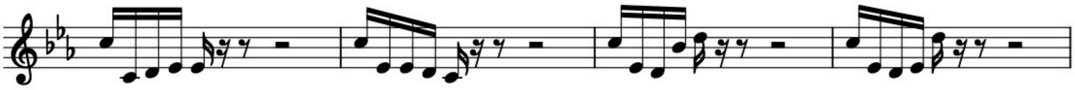

Same tone III

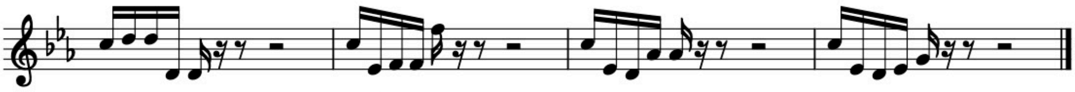

Melody 2

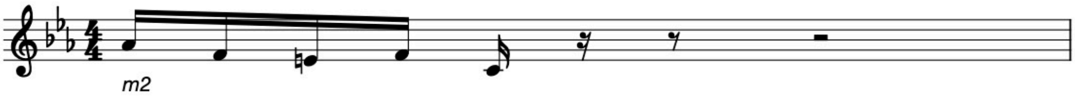

Inverted melodic contour I

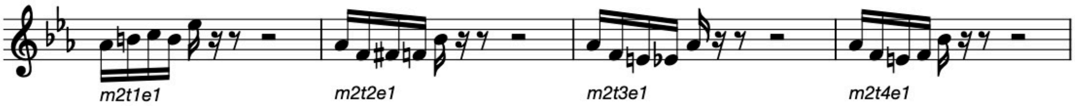

Same tone scrambled I

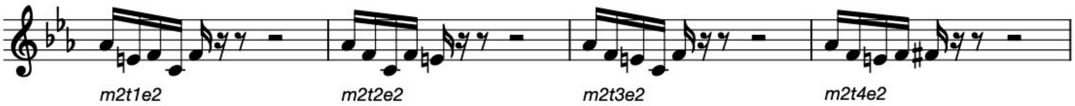

Same tone scrambled II

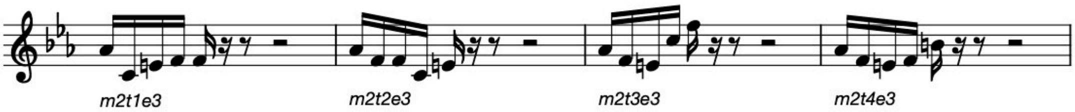

Same tone I

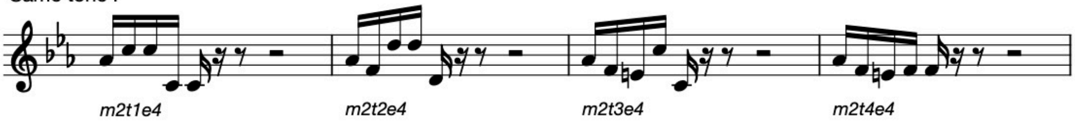

Same tone II

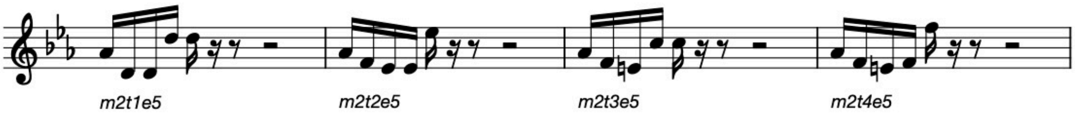

Scrambling intervals

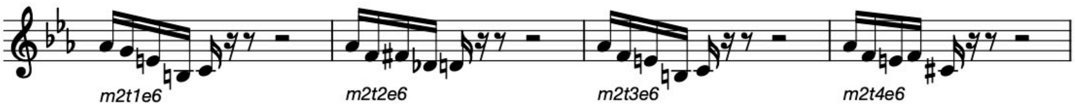

Inverted melodic contour II

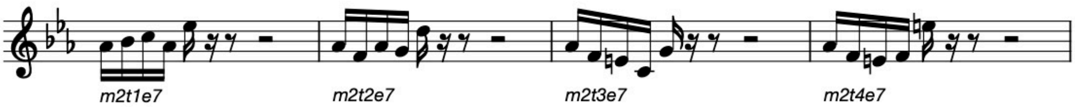

Same tone scrambled III

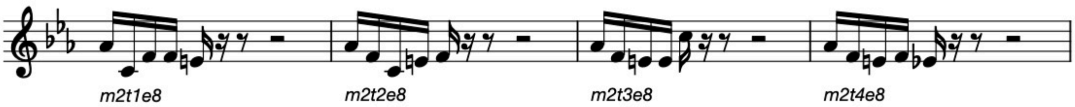

Same tone III

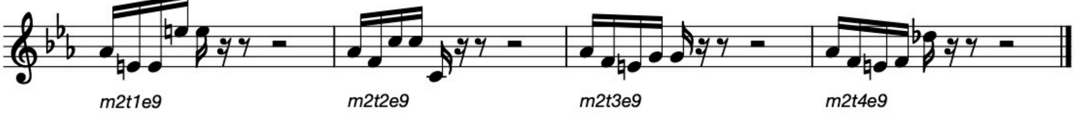

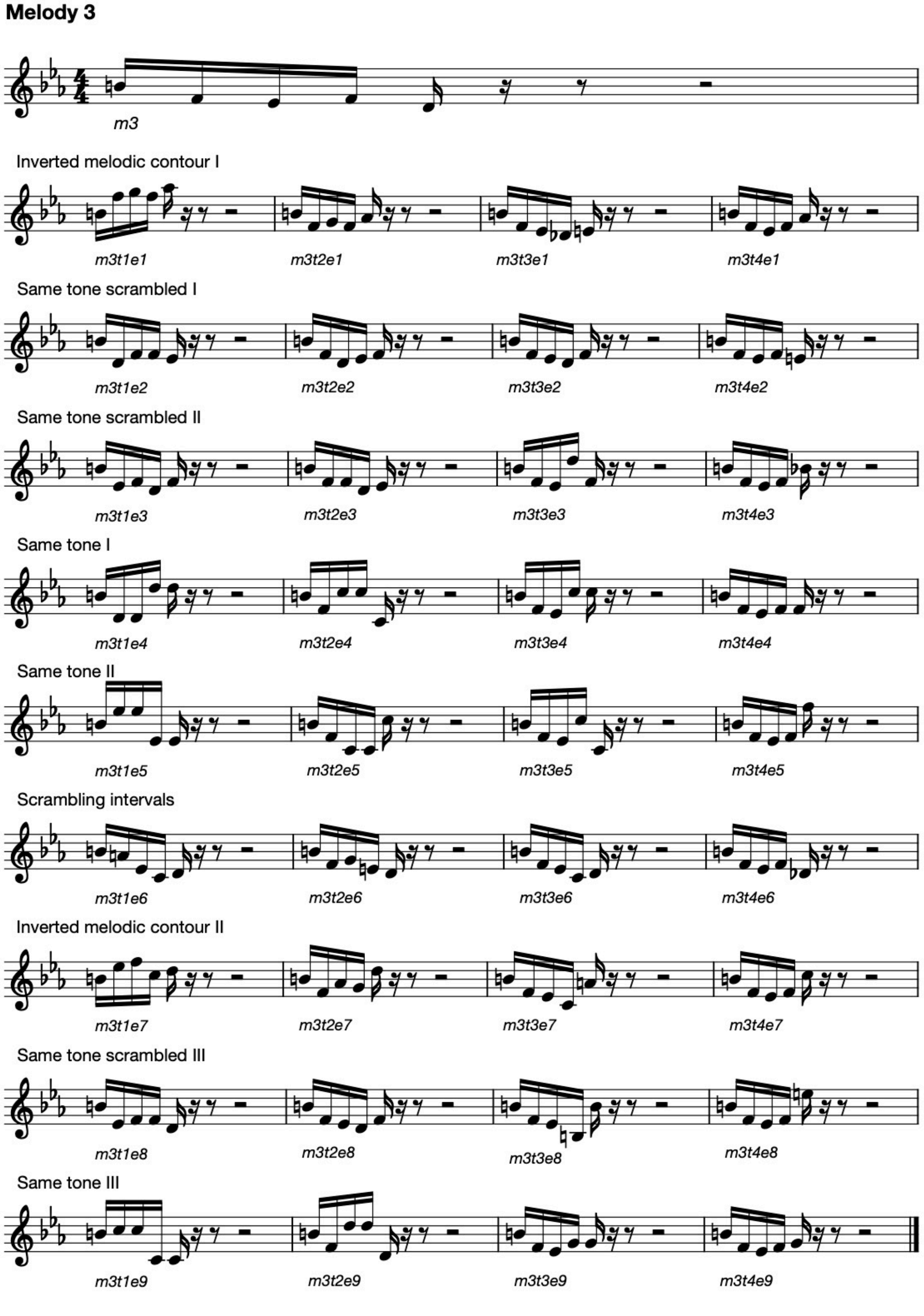

Figure S2. Auditory sequences used in the experiment

The figure presents all the auditory sequences utilised in the experiment in musical notation. The memorised (M) sequences consisted of three sequences, each comprising the first five notes of the first three bars of the musical piece. These sequences (m1, m2, and m3) were presented nine times each, resulting in a total of 27 trials. The novel (N)

sequences were generated through systematic variations of the three M sequences. This process involved altering every musical note in the sequence after the first (NT1), second (NT2), third (NT3), or fourth (NT4) note. Nine variations were created for each of the original M sequences across the four categories of N. This resulted in 27 N sequences for each category, totalling 108 N sequences. As illustrated in this figure, the variations were created according to the following rules: (i) Inverted melodic contours (used twice): the melodic contour of the variation was inverted in relation to the original M sequence (e.g., if the M sequence had the melodic contour: down-down-up-down, the N sequence would be: up-up-down-up); (ii) Same tone scrambled (used three times): the remaining notes of the M sequence were scrambled (e.g., M sequence: C-E-D-E-C was transformed into NT1 sequence: C-C-E-E-D). When this was not feasible (e.g., in the case of NT4, where only the last note differed from the M sequence), the last note of the M sequence was substituted with a random note; (iii) Same tone (used three times): the same note was repeatedly employed, sometimes varying only the octave (e.g., M sequence: C-E-D-E-C was changed to NT1 sequence: C-E8-E8-E8-E8); (iv) Scrambling intervals (used once): the intervals between the notes were scrambled (e.g., M sequence: 6thm – 2ndm – 2ndm – 3rdm was adapted to NT1 sequence: 2ndm, 6thm, 3rdm, 2ndm). In most cases, the harmonic structure of the N sequences in relation to the M sequences was preserved, with a few exceptions occurring for the 'inverted melodic contours' and 'scrambling intervals'. This strategy was employed to mitigate potential confounding variables arising from changes in harmony.

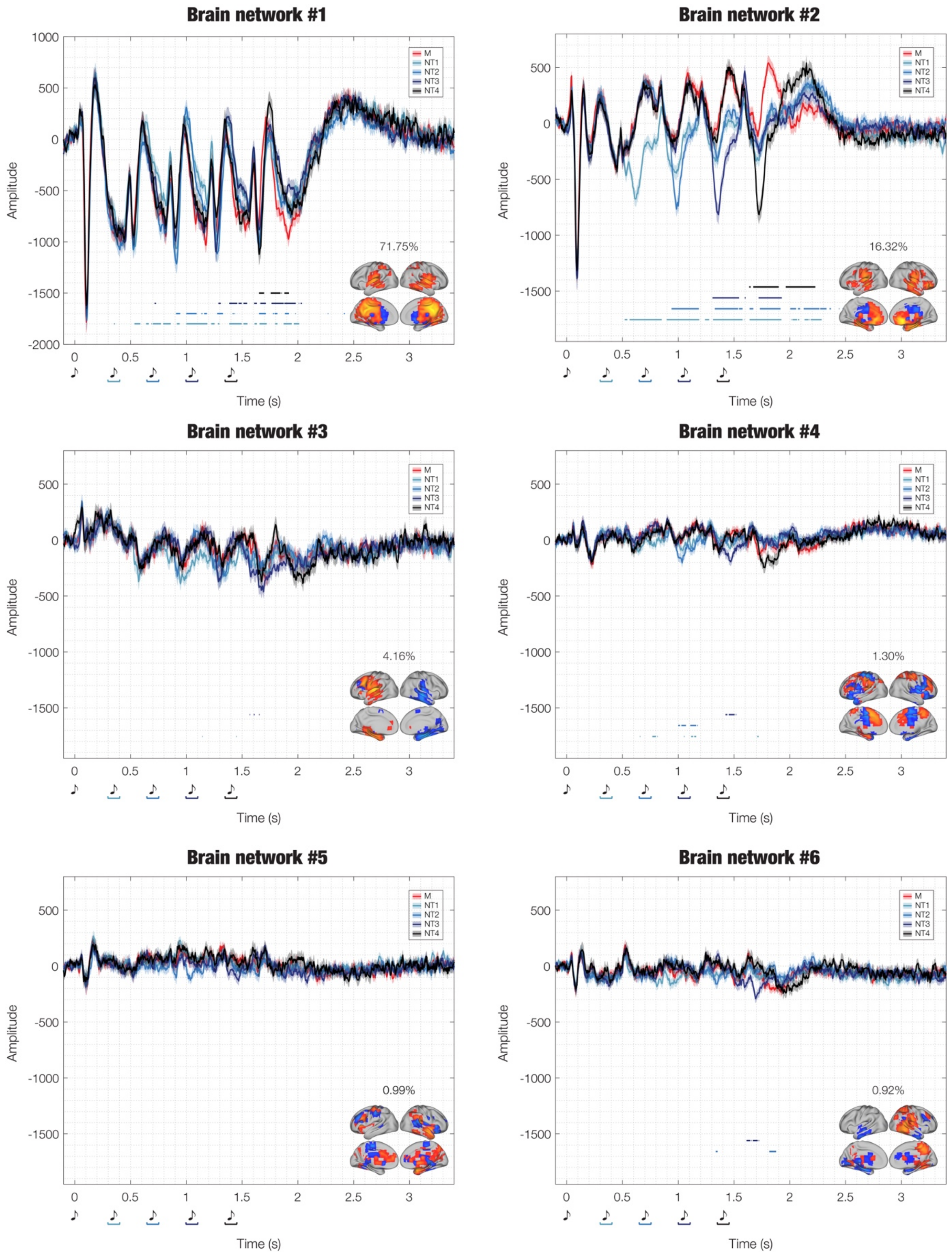

Figure S3. Time series and spatial activation patterns of significant brain networks according to the Monte-Carlo Simulations (MCS) approach

*This figure illustrates the time series and spatial activation patterns of brain networks that explained a higher variance than the first PCA component computed on time-wise randomised data (**Figure 3**), where both the order of brain voxels*

and time indices of the time series were disrupted. The variance explained by these surviving networks was: 71.75%, 16.32%, 4.16%, 1.30%, 0.99% and 0.92%, respectively. The figure shows that while six brain networks explained more variance than chance level (as determined by the randomised data), the first two brain networks accounted for most of the variance and displayed the clearest patterns in terms of differential brain activity between experimental conditions and spatial extent. As with the results shown in ***Figure 1****, independent time series for each participant, brain network, and experimental condition were generated using PCA-derived weights from the averaged data. The individual time series were then averaged across participants, as shown in the plots. Shaded areas represent standard errors. The brain templates illustrate the spatial extent of the networks, with yellow voxels yielding the most positive and light blue voxels yielding the most negative contribution to the time series. Only voxels with values exceeding the mean by more than one standard deviation in absolute terms are depicted. Blue-black lines indicate the temporal extent of the significant differences between M versus each category of N (i.e. M versus NT1, M versus NT2, M versus NT3, M versus NT4), computed using two-sided t-tests corrected for multiple comparisons via False Discovery Rate (FDR). Different shades of blue-black represent specific M versus N comparisons.*

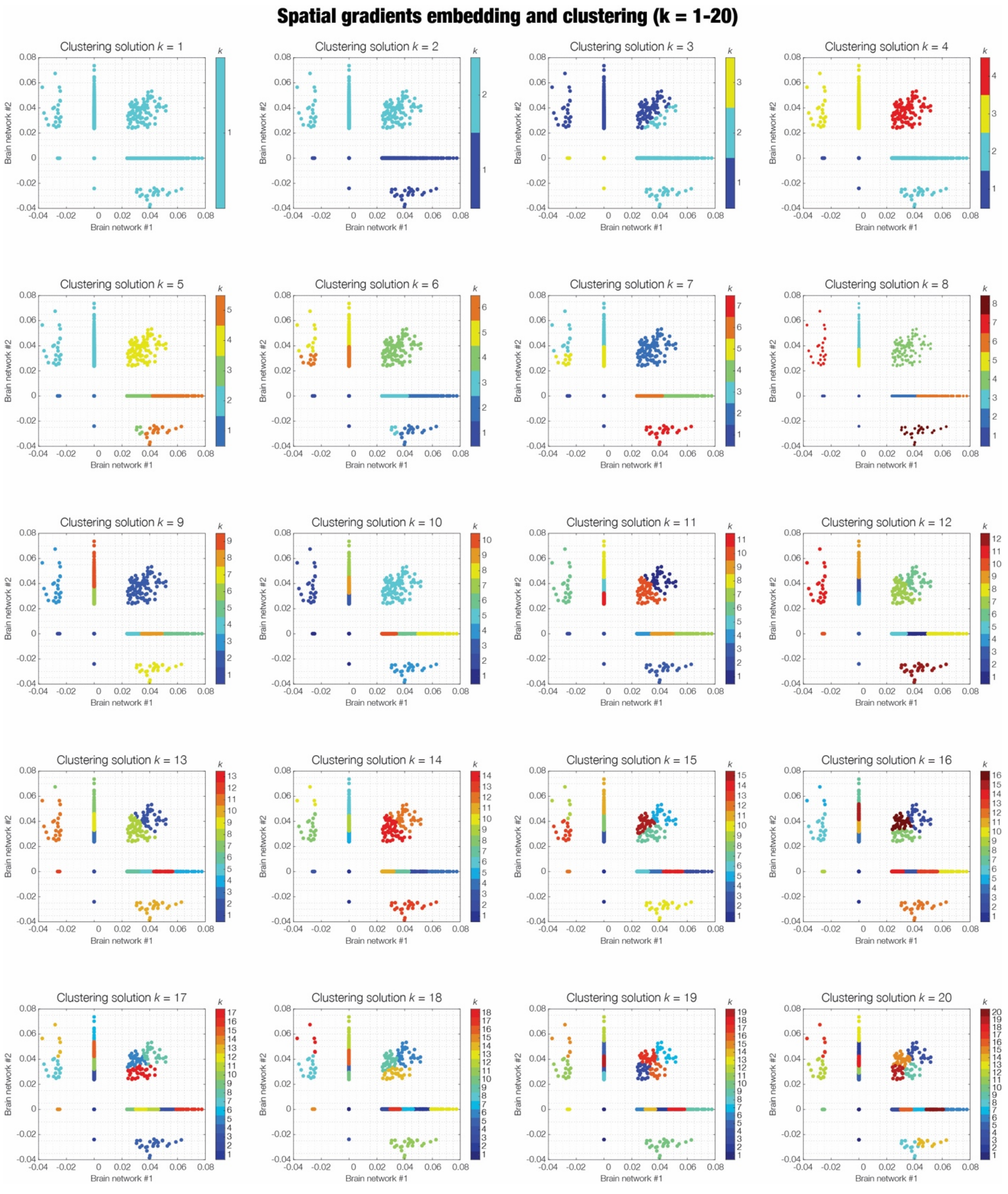

Figure S4. Voxel-wise clustering solutions for spatial gradients embedding of BROAD-NESS-derived brain networks (k = 1 to 20).

Each subplot shows a 2D embedding of brain voxels based on their contribution to the two principal BROAD-NESS networks, with colour-coded clusters derived from unsupervised k-means clustering for values of k ranging from 1 to 20. The embedding reflects how individual voxels co-participate across networks, revealing patterns of overlap, selectivity, or opposition. The full panel allows visual comparison across clustering solutions to identify consistent subdivisions of

the brain voxels across multiple runs of clustering analysis. The optimal number of clusters, determined through silhouette scores and repetition stability (corresponding to k = 8), is reported both here and in higher detail in the main text.

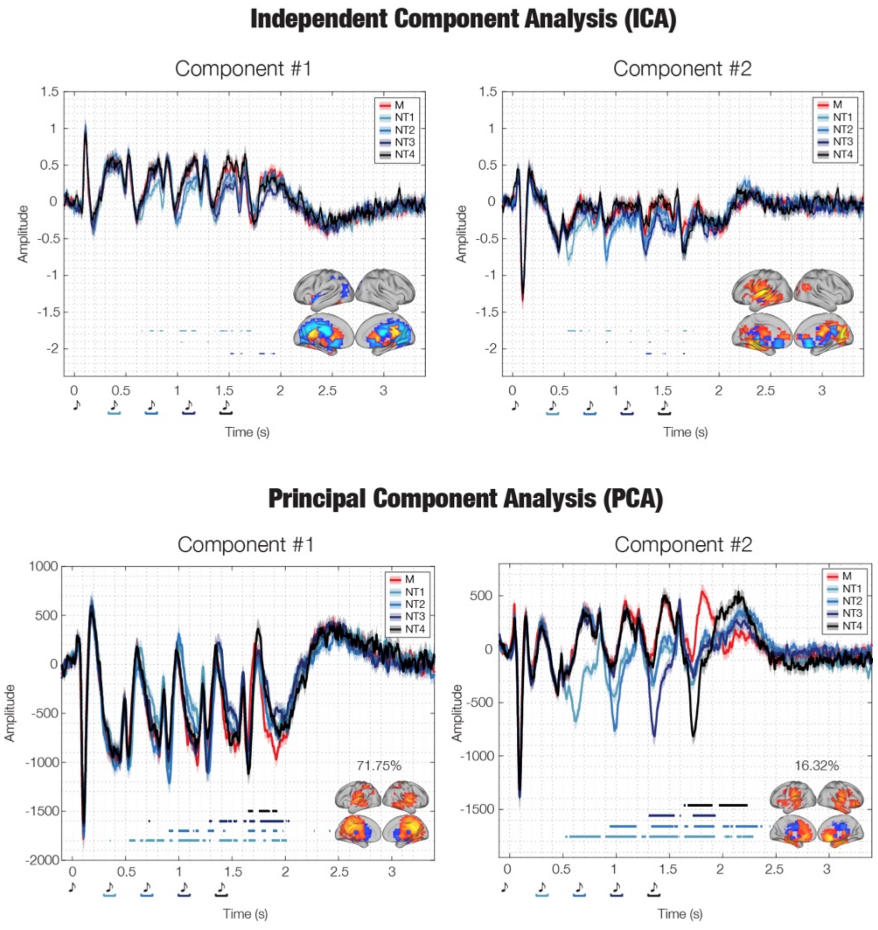

Figure S5. Comparison between first two BROAD-NESS-derived Principal Components (PCs) and Independent Components extracted using Independent Components Analysis (ICA, two-component decomposition).

Time series and spatial maps in brain templates (threshold for visualisation = mean plus one standard deviation) of the first two components extracted using ICA (top) and PCA (bottom) from source-reconstructed MEG data (n = 83 participants). ICA was computed using a two-component decomposition to match the key number of brain networks identified by BROAD-NESS. Each line in the time series plots represents the mean response per condition (M = memorised, NT1–NT4 = novel tone conditions), with shaded areas indicating the standard error of the mean. The brain templates illustrate the spatial extent of the networks, with yellow voxels yielding the most positive and light blue voxels yielding the most negative contribution to the time series. Only voxels with values exceeding the mean by more than one standard deviation in absolute terms are depicted. Horizontal bars mark statistically significant time points (two-sided t-tests, FDR-corrected, $p < .05$), with line colours indicating comparisons between M and each novel condition (NT1 = light blue, NT2 = blue, NT3 = dark blue, NT4 = black). While both ICA and PCA captured some task-related dynamics, the PCA-derived components (accounting for 71.75% and 16.32% of the variance, respectively) displayed clearer temporal structure, stronger condition sensitivity, and more interpretable spatial organisation. These results highlight the improved interpretability and variance-based ranking offered by the BROAD-NESS PCA approach relative to ICA when applied to the same dataset.

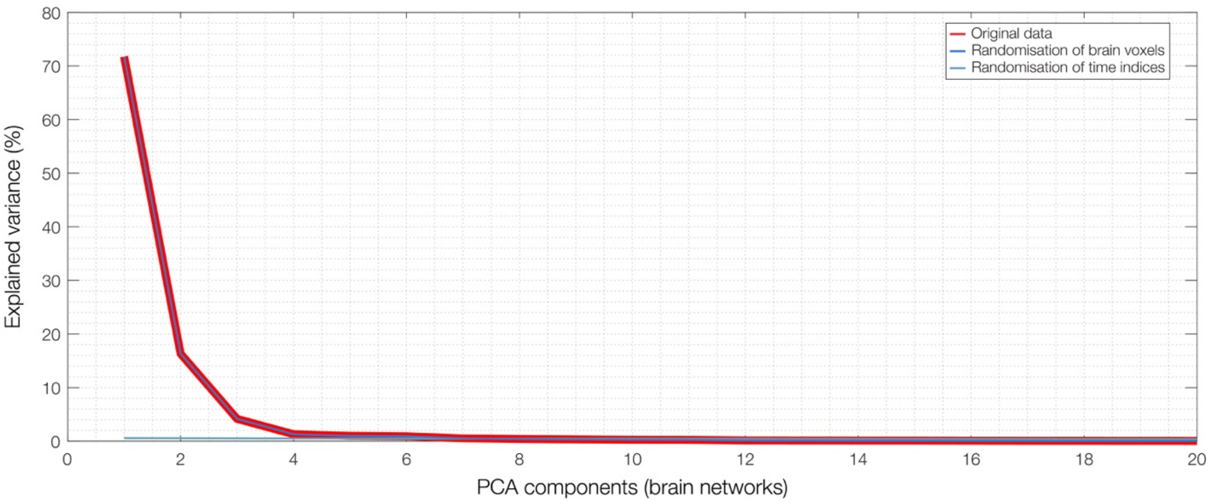

Figure S6. Variance explained by the top 20 PCA components for the original and randomised data

This figure illustrates the variance accounted for by the PCA components following analysis of the original data compared to the randomised data (both space randomisation and time randomisation). The results demonstrate that the variance explained by the original data and the space-randomised data remains identical. As shown in Figure 3, this randomisation impacted only the spatial activation patterns, without affecting the variance explained or the time series. In contrast, time randomisation led to a significant reduction in the variance explained by the top PCA components, alongside a disruption of both the spatial activation patterns of the brain networks and the time series.

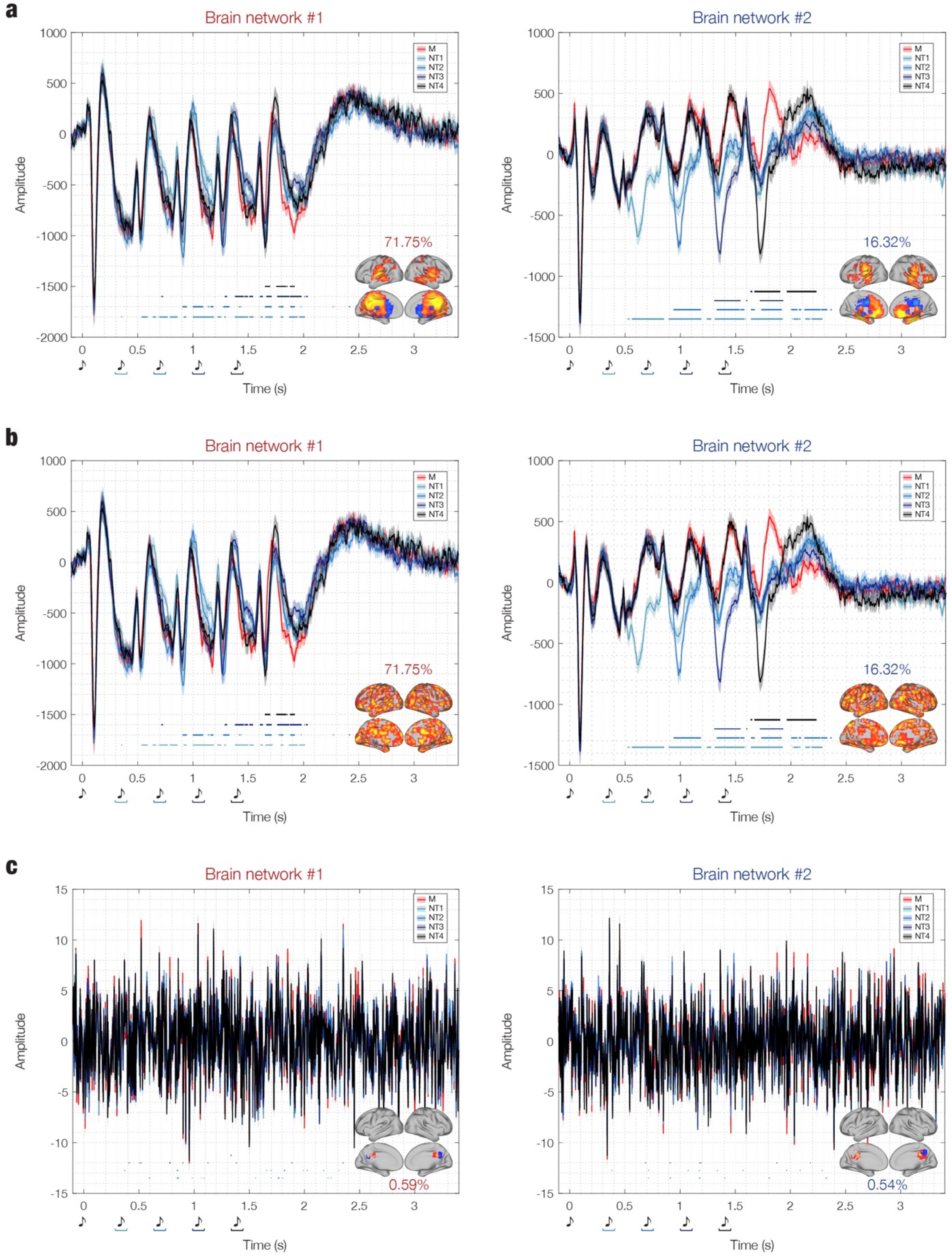

Figure S7. Brain networks estimated from randomised data

This figure illustrates the impact of data randomisation on the estimation of brain networks. ***a -*** *Brain networks computed from the original data, identical to those shown in* ***Figure 2****.* ***b -*** *Space randomisation, where the order of the brain voxels was randomised without altering their time series. The time series and explained variance remain the same as in the original data, but the spatial activation patterns are scrambled. This demonstrates that PCA is independent of voxel order, confirming that meaningful patterns in the brain networks are genuine and not artefacts of the PCA algorithm.* ***c***

*– Time randomisation, where the time indices of the time series were disrupted, independently for each brain voxel. This leads to meaningless, low-amplitude time series and scrambled activation patterns, mostly concentrated around the mean. As a result, only a few brain voxels are depicted in the templates. In all cases, independent time series for each participant, brain network, and experimental condition were generated using PCA-derived weights from the averaged data. The individual time series were then averaged across participants, as shown in the plots. Shaded areas represent standard errors. The brain templates illustrate the spatial extent of the networks, with yellow voxels yielding the most positive and light blue voxels yielding the most negative contribution to the time series. Only voxels with values exceeding the mean by more than one standard deviation in absolute terms are depicted. Blue-black lines indicate the temporal extent of the significant differences between M versus each category of N (i.e. M versus NT1, M versus NT2, M versus NT3, M versus NT4), computed using two-sided t-tests corrected for multiple comparisons via False Discovery Rate (FDR). Different shades of blue-black represent specific M versus N comparisons. The variance explained by the PCs for the original data and the two randomisation strategies is depicted in **Figure S6**.*

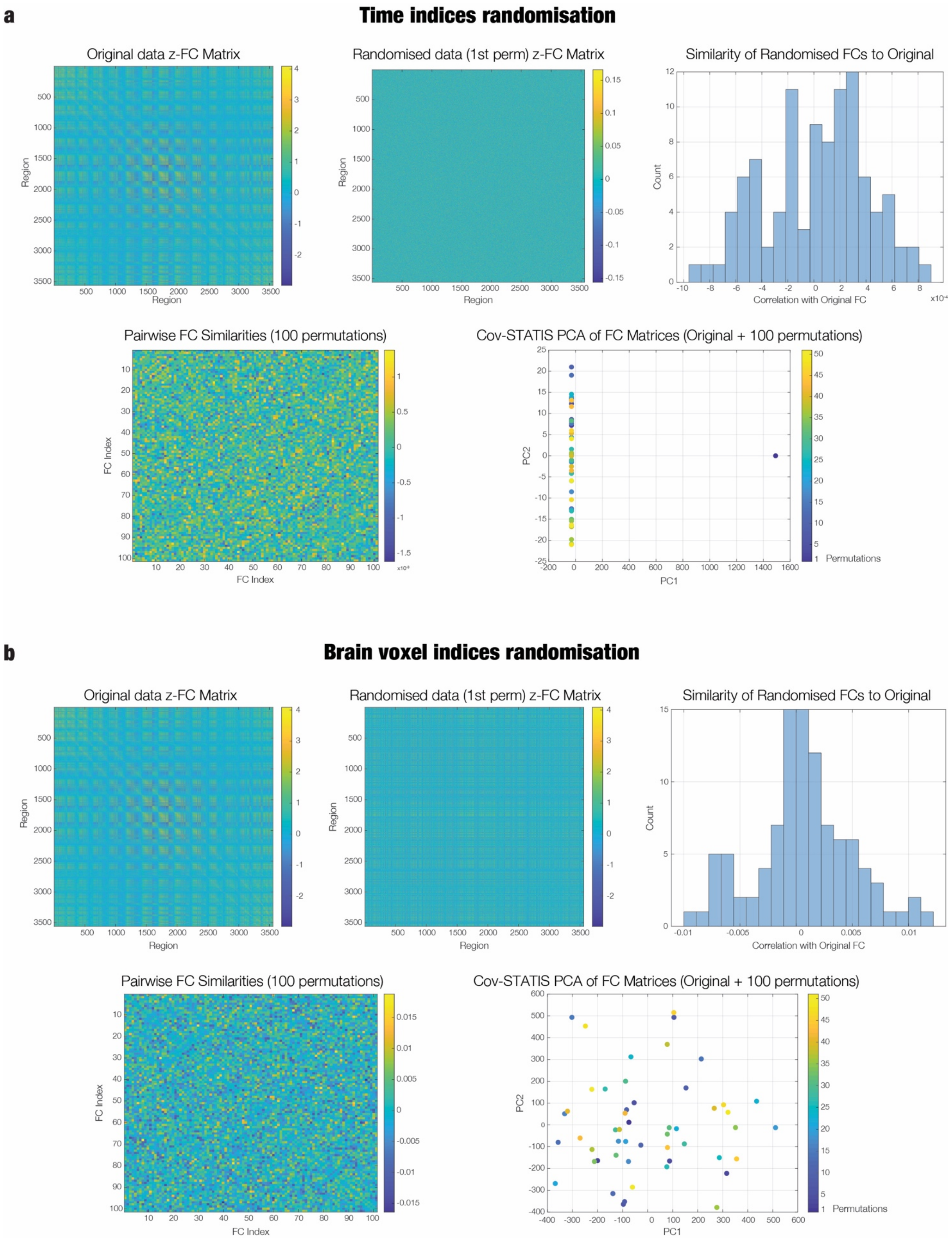

Figure S8. Functional connectivity disruption through time and space randomisation.

Validation analyses assessing the effectiveness of BROAD-NESS randomisation procedures in disrupting functional connectivity (FC) patterns. ***a*** *- Time index randomisation. Left to right: 1) FC matrix from original data (Pearson correlation of time series between all brain voxels); 2) FC matrix from the randomised dataset (permutation #1), where*

time indices were independently shuffled across voxels; 3) histogram of similarity (Pearson correlation) between the original FC and 100 permuted FCs; 4) matrix of pairwise similarities between all FC matrices (original + 100 permutations); 5) PCA on the covariance structure of FC matrices (Cov-STATIS) showing that the original FC is clearly separable from permuted datasets. ***b -*** *Voxel index randomisation. Same layout as in panel* ***a)*** *but using spatial (voxel index) randomisation instead of temporal shuffling. While voxel permutation preserves some spatial features when computing static FC (because the time series were not disrupted but only the spatial order of the brain voxels), both procedures effectively degrade the structure of the original FC, as confirmed by the drop in similarity and separation in PCA space. These analyses confirm that BROAD-NESS randomisation successfully disrupts spatiotemporal dependencies in the data, exactly as intended.*

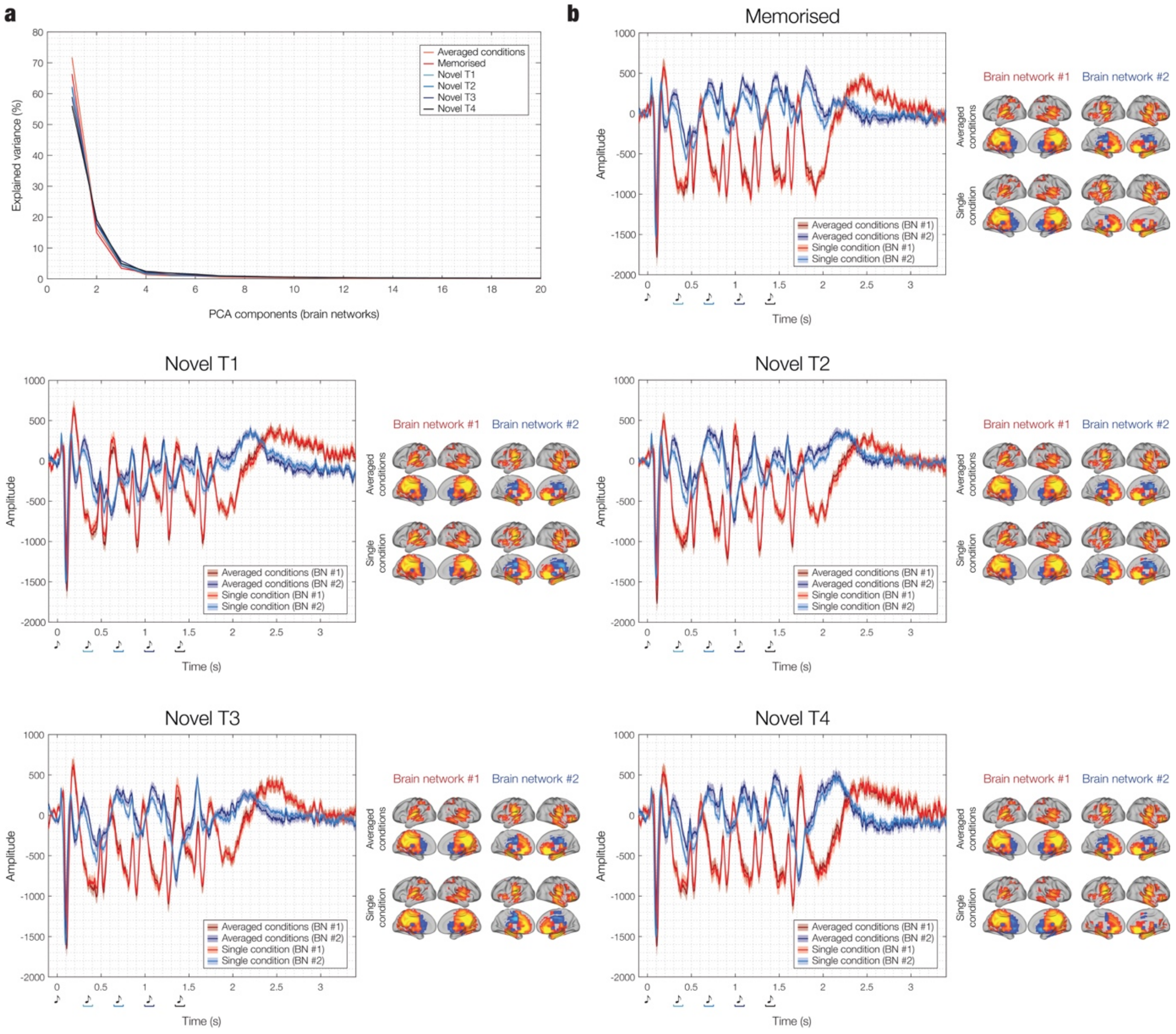

Figure S9. Brain networks estimated from data averaged across conditions or independently for each condition

***a -** Variance explained by the PCs after performing PCA on data averaged across conditions or independently for each condition. The nearly equivalent variance suggests that the different experimental conditions are linked to the same brain networks. **b -** Comparison of the time series for the two brain networks, computed using PCA weights from either averaged data or data selected independently for each condition. The time series are nearly identical, further supporting the notion that the brain networks underlying the different experimental conditions are extremely similar. Indeed, the differences between experimental conditions arise not in the brain networks spatial extent, but rather in the polarity and temporal dimension of their responses. In all cases, independent time series for each participant, brain network, and experimental condition were generated using PCA-derived weights from the data averaged across participants. The individual time series were then averaged across participants, as shown in the plots. Shaded areas represent standard errors. The brain templates illustrate the spatial extent of the networks, with yellow voxels yielding the most positive and light blue voxels yielding the most negative contribution to the time series. Only voxels with values exceeding the mean by more than one standard deviation in absolute terms are depicted.*

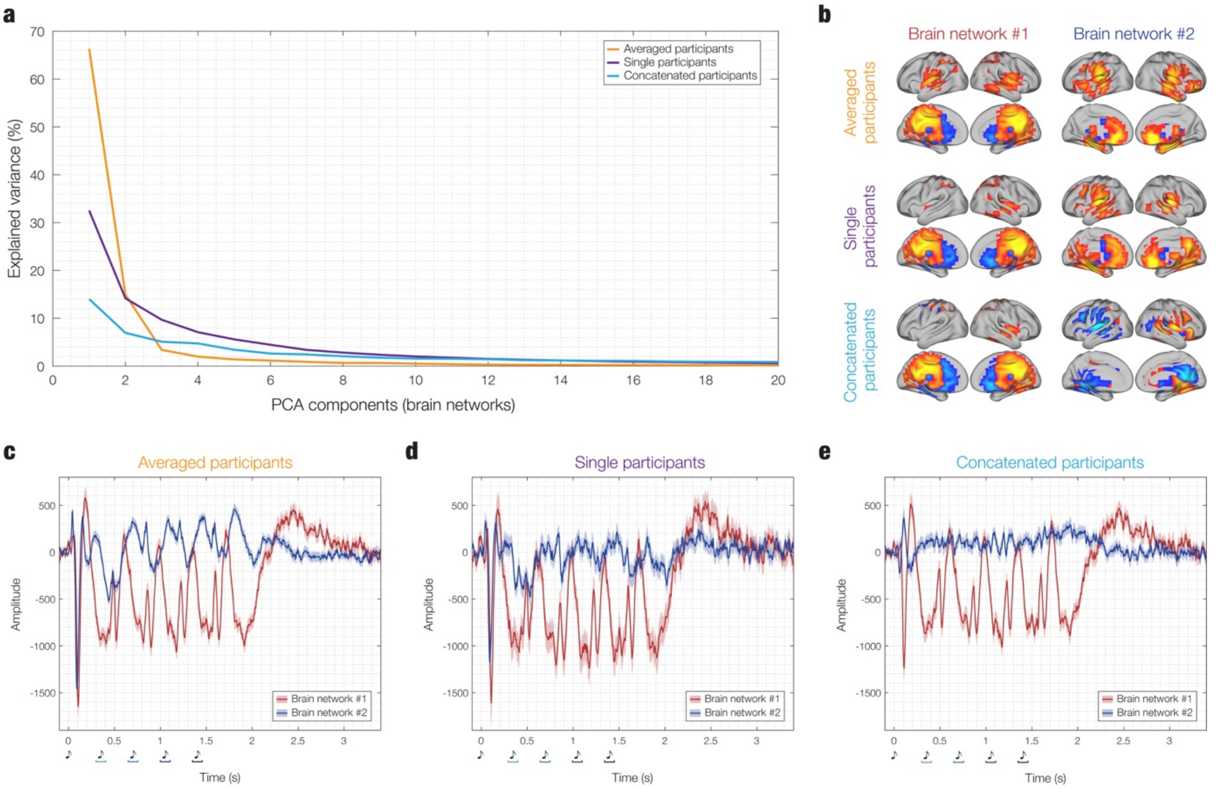

Figure S10. Brain networks estimated from data averaged across participants or independently for each participant

***a** - Variance explained by the PCs after performing PCA on data averaged across participants, or independently for each participant, considering one experimental condition: the previously memorised sequences. The results show a clear reduction in the variance explained by the first components when PCA is performed independently on individual participants (and the results averaged) or on the concatenated data from all participants, compared to PCA computed on data averaged across participants. **b** - The spatial activation patterns are depicted in brain templates, with yellow voxels contributing the most and light blue voxels the least to the corresponding time series. Only voxels with values exceeding the mean by more than one standard deviation in absolute terms are depicted. The plots show that the spatial activation patterns of the first brain network remain relatively stable, despite the drop in variance observed in **a**. However, the spatial activation patterns for the second brain network show a clear shift. When PCA is computed for individual participants (and results averaged), the network is less clearly defined but still maintains a similar spatial extent. In contrast, when PCA is performed on the concatenated data from all participants, the spatial extent of the second network drastically changes, losing its relevance. **c** - The time series were generated in three different ways. In the first case, independent time series for each participant and brain network were computed using PCA-derived weights from data averaged across participants. In the second case, PCA was computed independently for each participant, and individual time series were generated using participant-specific PCA weights. In the third case, PCA was computed on the concatenated data from all participants, and those weights were used to independently generate time series for each participant. In all cases, the individual time series were subsequently averaged across participants, as shown in the plots. Shaded areas represent standard errors. Overall, the time series results are consistent with the spatial activation patterns. The first brain network remains relatively stable across the different methods of computation, while the second network shows substantial changes. Specifically, the time series becomes less well-defined in the individual-participant computation and approaches a flat line in the concatenated data computation. This suggests that PCA should be performed on data averaged across participants, and the resulting weights should be used to compute the time series independently for each participant and experimental condition.*

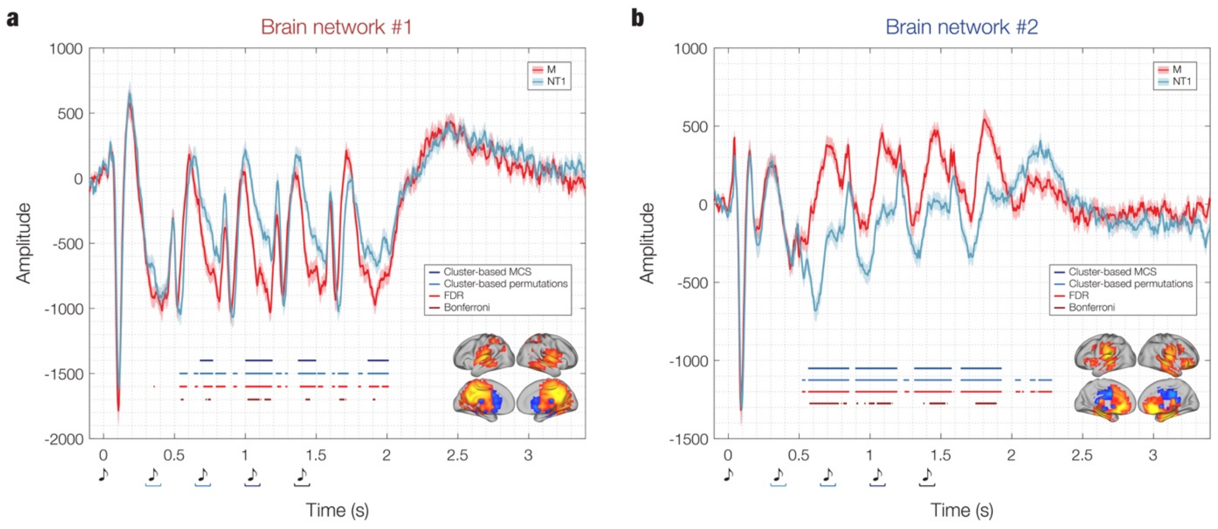

Figure S11. Multiple comparisons corrections

This figure presents an empirical comparison of four methods for correcting multiple comparisons.

*First, the plots illustrate the time series averaged across participants for each brain network and two experimental conditions (previously memorised and Novel T1 [NT1] sequences). Shaded areas represent standard errors. The brain templates illustrate the spatial extent of the networks, with yellow voxels contributing the most and light blue voxels contributing the least to the time series. Only voxels with values exceeding the mean by more than one standard deviation in absolute terms are depicted. Second, the plots highlight significant differences that survived various correction methods after contrasting previously memorised with NT1 sequences using two-sided t-tests for each time point and brain network. The methods compared are: (i) Bonferroni, (ii) False Discovery Rate (FDR), (iii) Cluster-based permutation test, and (iv) Cluster-based Monte Carlo Simulations (MCS). Bonferroni correction adjusts the significance level (α = .05) by dividing it by the number of tests performed, thereby reducing the likelihood of false positives. FDR ranks p-values from multiple tests, comparing them to the alpha level (α = .05) and identifying a stricter threshold (q) to control the proportion of false discoveries. The cluster-based permutation test, as described by Maris and Oostenveld[xxviii], conducts statistical tests on the original data, identifies clusters of significant time points (α = .05), and performs 1000 permutations to shuffle labels and recalculate statistics. Clusters are deemed significant if their sizes exceed those from permuted data 99.9% of the time. Cluster-based MCS (MCS, α = .05, MCS p-value = .001), as detailed in our previous studies***Error! Bookmark not defined.***[,xxix-xxxv], identifies clusters of significant neighbouring time points (α = .05), performs 1000 permutations to randomise these values, and considers the clusters from the original data significant if they exceed 99.9% of the sizes of the ones obtained from the permuted data. The convergence of results across these methods further reinforces the robustness of the findings.*

Supplementary Tables

The supplementary tables are available at the following link:
https://doi.org/10.5281/zenodo.17048137

Table S1. Statistical analysis of the two main brain networks
This table presents the results of statistical analyses conducted independently for the two brain networks that explained the highest variance. At each time point, t-tests compared memorised (M) versus each category of novel (NTs) sequences (i.e., M versus NT1, M versus NT2, M versus NT3, M versus NT4). Multiple comparisons were corrected using the false discovery rate (FDR) method. The table highlights significant time points along with the corresponding t-values.

Table S2. MNI coordinates of brain voxels assigned to each cluster in the spatial gradient embedding analysis
This table reports the results of the voxel-wise clustering analysis based on the spatial gradient embedding of the two principal BROAD-NESS networks. Voxels were clustered according to their contributions to Network #1 and Network #2 (represented by PCA components PC1 and PC2, respectively), using an unsupervised k-means clustering approach. The optimal solution identified eight clusters, each shown in a separate sheet. For each voxel, the table provides its progressive index and MNI coordinates (X, Y, Z), along with its corresponding values (spatial activation pattern) on PC1 and PC2.

Table S3. Correlation between brain network activity and behavioural measures
This table presents the results of correlation analyses between the time series of the two main brain networks and behavioural measures, including accuracy, reaction times (RTs), and musical expertise. Analyses were conducted separately for each network and behavioural variable, across time. Multiple comparisons were corrected using the false discovery rate (FDR) method. The table reports the time points with significant correlations, along with the corresponding correlation coefficients.

Table S4. Statistical analysis of independent component analysis (ICA) results
This table presents the results of statistical analyses performed on brain networks obtained via independent component analysis (ICA). Two ICA decompositions were conducted: one with 14 components and one with two components, for comparison with the PCA-based networks. For each decomposition and ICA component (brain network) time series, t-tests compared memorised (M) sequences with each category of novel (NTs) sequences (i.e., M versus NT1, M versus NT2, M versus NT3, M versus NT4) across time. Multiple comparisons were corrected using the false discovery rate (FDR) method. The table reports the time points with significant differences for each ICA component (brain network) and comparison, as well as the correspondent p-values and t-values.

Table S5. Statistical analysis of the two main brain networks obtained after randomisation of the data
This table presents the results of statistical analyses conducted independently for the two brain networks that explained the highest variance, separately for the two randomisation strategies adopted here: space randomisation and time randomisation. At each time point, t-tests compared memorised (M) versus each category of novel (NTs) sequences (i.e.,

M versus NT1, M versus NT2, M versus NT3, M versus NT4). Multiple comparisons were corrected using the false discovery rate (FDR) method. Significant time points and their corresponding t-values are reported.

Table S6. Comparison of significant results after different solutions for multiple comparisons correction

This table presents the empirical comparison of four methods for multiple comparisons correction. First, t-tests were conducted independently for each time point across the two main brain networks, contrasting memorised (M) versus each category of novel (NTs) sequences (i.e., M versus NT1, M versus NT2, M versus NT3, M versus NT4). Second, four different methods for multiple comparisons correction were employed: (i) Bonferroni, (ii) False Discovery Rate (FDR), (iii) cluster-based permutation test, and (iv) cluster-based Monte Carlo simulations (MCS). For Bonferroni and FDR corrections, the table displays significant time points along with their corresponding t-values. Regarding the cluster-based methods, significant clusters are reported, including cluster size, p-value, temporal extent of the cluster, and peak t-value within each cluster. These results are illustrated in ***Figure S11****.*

References

[i] Nieto-Castanon, A., Ghosh, S. S., Tourville, J. A. & Guenther, F. H. Region of interest based analysis of functional imaging data. *Neuroimage* **19**, 1303–1316 (2003). https://doi.org/10.1016/S1053-8119(03)00188-5

[ii] Padilla-Buritica, J. I., Martinez-Vargas, J. D. & Castellanos-Dominguez, G. Emotion discrimination using spatially compact regions of interest extracted from imaging EEG activity. *Front. Comp. Neurosci.* **10**, (2016). https://doi.org/10.3389/fncom.2016.00055

[iii] Mamashli, F., Hämäläinen, M., Ahveninen, J. *et al.* Permutation statistics for connectivity analysis between regions of interest in EEG and MEG Data. *Sci. Rep.* **9**, 7942 (2019). https://doi.org/10.1038/s41598-019-44403-z

[iv] Marwan, N., Wessel, N., Meyerfeldt, U., Schirdewan, A. & Kurths, J. Recurrence-plot-based measures of complexity and their application to heart-rate-variability data. *Phys Rev E Stat Phys Plasmas Fluids Relat Interdiscip Topics* **66**, (2002). https://doi.org/10.1103/PhysRevE.66.026702

[v] Marwan, N., Carmenromano, M., Thiel, M. & Kurths, J. Recurrence plots for the analysis of complex systems. *Phys. Rep.* **438**, 237–329 (2007). https://doi.org/10.1016/j.physrep.2006.11.001

[vi] Vicente, R., Wibral, M., Lindner, M. & Pipa, G. Transfer entropy—a model-free measure of effective connectivity for the neurosciences. *J. Comput. Neurosci.* **30**, 45–67. (2010). https://doi.org/10.1007/s10827-010-0262-3

[vii] Friston, K., Harrison, L., & Penny, W. Dynamic causal modelling. *Neuroimage,* **19**(4), 1273–1302. (2003). https://doi.org/10.1016/s1053-8119(03)00202-7

[viii] Vidaurre, D., Smith, S. M., & Woolrich, M. W. (2017). Brain network dynamics are hierarchically organized in time. *Proceedings of the National Academy of Sciences*, **114(**48), 12827–12832. https://doi.org/10.1073/pnas.1705120114

[ix] Ahrends, C., Woolrich, M. W., & Vidaurre, D. Predicting individual traits from models of brain dynamics accurately and reliably using the Fisher kernel. *eLife*, **13** (2025). https://doi.org/10.7554/elife.95125.3

[x] Cabral, J., Vidaurre, D., Marques, P. *et al.* Cognitive performance in healthy older adults relates to spontaneous switching between states of functional connectivity during rest. *Sci Rep* **7**, 5135 (2017). https://doi.org/10.1038/s41598-017-05425-7

[xi] Marwan, N., Carmenromano, M., Thiel, M. & Kurths, J. Recurrence plots for the analysis of complex systems. *Phys. Rep.* **438**, 237–329 (2007). https://doi.org/10.1016/j.physrep.2006.11.001

[xii] Bradley, E. & Kantz, H. Nonlinear time-series analysis revisited. *Chaos* **25** (2015). https://doi.org/10.1063/1.4917289

[xiii] Ding, M., Chen, Y. and Bressler, S. L. Granger Causality: Basic Theory and Application to Neuroscience. In *Handbook of Time Series Analysis* (eds B. Schelter, M. Winterhalder and J. Timmer). (Wiley-VCH, New Jersey, 2006). https://doi.org/10.1002/9783527609970.ch17

[xiv] Ruchkin, D. S., Villegas, J. & John, E. R. An analysis of average evoked potentials making use of least mean square techniques. *Ann. N. Y. Acad. Sci.* **115**, 799–826 (1964). https://doi.org/10.1111/j.1749-6632.1964.tb50665.x

[xv] Picton, T. W. *et al.* Guidelines for using human event-related potentials to study cognition: Recording standards and publication criteria. *Psychophysiology* **37**, 127–152 (2000). https://doi.org/10.1111/1469-8986.3720127

[xvi] Dien J, & Frishkoff, G. A. *Principal components analysis of event-related potential datasets. In: Event-related potentials: a methods handbook* (Handy T. C., ed), pp 189–208. (Cambridge, MIT Press, 2005).

[xvii] Dien, J. Applying principal components analysis to event-related potentials: A tutorial. *Dev. Neuropsychol.* **37**, 497–517 (2012). https://doi.org/10.1080/87565641.2012.697503

[xviii] Donchin, E. A multivariate approach to the analysis of average evoked potentials. *IEEE Trans. Biomed. Eng.* **13**, 131–139 (1966). https://doi.org/10.1109/tbme.1966.4502423

[xix] Duffy, F. H., Jones, K., Bartels, P., McAnulty, G. & Albert, M. Unrestricted principal components analysis of brain electrical activity: Issues of data dimensionality, artifact, and utility. *Brain Topogr.* **4**, 291–307 (1992). https://doi.org/10.1007/bf01135567

[xx] Kayser, J., & Tenke, C. E. Optimizing PCA methodology for ERP component identification and measurement: theoretical rationale and empirical evaluation. *Clin. Neurophysiol. Pract.* **114**(12), 2307–2325. (2003). https://doi.org/10.1016/s1388-2457(03)00241-4

[xxi] McKeown, M. J. & Sejnowski, T. J. Independent component analysis of fMRI data: examining the assumptions. *Hum. Brain Mapp.* **6**, 368–372 (1998). https://doi.org/10.1002/(SICI)1097-0193(1998)6:5/6<368::AID-HBM7>3.0.CO;2-E

[xxii] Zhong, Y. *et al.* Detecting functional connectivity in fMRI using PCA and regression analysis. *Brain Topogr.* **22**, 134–144. (2009). https://doi.org/10.1007/s10548-009-0095-4

[xxiii] Carbonell, F., Bellec, P. & Shmuel, A. Global and system-specific resting-state fMRI fluctuations are uncorrelated: principal component analysis reveals anti-correlated networks. *Brain Connect*. **1**, 496–510. (2011). https://doi.org/10.1089/brain.2011.0065

[xxiv] Leonardi, N. *et al.* Principal components of functional connectivity: a new approach to study dynamic brain connectivity during rest. *Neuroimage* **83**, 937–950 (2013). https://doi.org/10.1016/j.neuroimage.2013.07.019

[xxv] McKeown, M. J. *et al.* Spatially independent activity patterns in functional MRI data during the Stroop color-naming task. *PNAS* **95**, 803–810 (1998). https://doi.org/10.1073/pnas.95.3.803

[xxvi] Scharf, F., Widmann, A., Bonmassar, C. & Wetzel, N. A tutorial on the use of temporal principal component analysis in developmental ERP research – Opportunities and challenges. *Dev. Cogn. Neurosci.* **54**, 101072 (2022). https://doi.org/10.1016/j.dcn.2022.101072

[xxvii] Rosso, M. *et al.* FREQ-NESS reveals the dynamic reconfiguration of frequency-resolved brain networks during auditory stimulation. *Adv. Sci.* (in press). Preprint at https://doi.org/10.1101/2024.08.28.610155 (2024).

[xxviii] Maris, E. & Oostenveld, R. Nonparametric statistical testing of EEG- and MEG-data. *J. Neurosci. Methods* **164**, 177–190 (2007). https://doi.org/10.1016/j.jneumeth.2007.03.024

[xxix] Bonetti, L., Fernández-Rubio, G., Lumaca, M. *et al.* Age-related neural changes underlying long-term recognition of musical sequences. *Commun Biol* **7**, 1036 (2024). https://doi.org/10.1038/s42003-024-06587-7

[xxx] Bonetti, L. *et al.* Spatiotemporal whole-brain activity and functional connectivity of melodies recognition. *Cerebr. Cortex* **34**, (2024). https://doi.org/10.1093/cercor/bhae320

[xxxi] Bonetti, L. *et al.* Brain recognition of previously learned versus novel temporal sequences: a differential simultaneous processing. *Cerebr. Cortex* **33**, 5524–5537 (2022). https://doi.org/10.1093/cercor/bhac439

[xxxii] Fernández-Rubio, G., Brattico, E., Kotz, S.A. *et al.* Magnetoencephalography recordings reveal the spatiotemporal dynamics of recognition memory for complex versus simple auditory sequences. *Commun. Biol.* **5**, 1272 (2022). https://doi.org/10.1038/s42003-022-04217-8

[xxxiii] Fernández-Rubio, G., Carlomagno, F., Vuust, P., Kringelbach, M. L. & Bonetti, L. Associations between abstract working memory abilities and brain activity underlying long-term recognition of auditory sequences. *PNAS Nexus* 1, (2022). https://doi.org/10.1093/pnasnexus/pgac216

[xxxiv] Hoegholt, N. F. *et al.* A magnetoencephalography study of first-time mothers listening to infant cries. *Cerebr. Cortex* **33**, 5896–5905 (2022). https://doi.org/10.1093/cercor/bhac469

[xxxv] Bonetti, L. *et al.* Moderate associations between BDNF Val66Met gene polymorphism, musical expertise, and mismatch negativity. *Heliyon* **9**, e15600 (2023). https://doi.org/10.1016/j.heliyon.2023.e15600
